# Genetic Architecture and Anthocyanin Profiling of Aromatic Rice From Manipur Reveals Divergence of *Chakhao* Landraces

**DOI:** 10.3389/fgene.2020.570731

**Published:** 2020-10-15

**Authors:** S. Bhuvaneswari, S. Gopala Krishnan, Haritha Bollinedi, Supradip Saha, Ranjith Kumar Ellur, K. K. Vinod, I. Meghachandra Singh, Narendra Prakash, Prolay Kumar Bhowmick, M. Nagarajan, Nagendra Kumar Singh, Ashok Kumar Singh

**Affiliations:** ^1^Division of Genetics, Indian Council of Agricultural Research (ICAR)-Indian Agricultural Research Institute, New Delhi, India; ^2^ICAR-Research Complex for North Eastern Hill Region, Manipur Centre, Imphal, India; ^3^Division of Agricultural Chemicals, ICAR-Indian Agricultural Research Institute, New Delhi, India; ^4^Rice Breeding and Genetics Research Centre, ICAR-Indian Agricultural Research Institute, Aduthurai, India; ^5^ICAR-National Institute of Plant Biotechnology, New Delhi, India

**Keywords:** Manipur black rice, population structure, antioxidant properties, SSR markers, anthocyanin, *Chakhao* landraces, diversity

## Abstract

Aromatic rice of Manipur popularly known as *Chakhao* is a speciality glutinous rice, for which protection under geographical indication in India has been granted recently. The agronomic and nutraceutical variability of the *Chakhao* rice germplasm is yet to be genetically characterized. To address this gap, characterization of ninety-three landraces for agro-morphological traits, grain pigmentation, antioxidant properties, and molecular genetic variation was carried out to unravel their population genetic structure. Two major groups were identified based on pericarp color, namely, purple and non-purple, which showed a significant variation for plant height, panicle length, and grain yield. Molecular marker analysis revealed three subpopulations that could be associated with pericarp pigmentation. Deep purple genotypes formed POP3, *japonica* genotypes adapted to hill environment formed POP1, while POP2 comprised of both *indica* and *aus* types. Liquid chromatography–mass spectrometry (LC-MS) analysis revealed two major anthocyanin compounds in pigmented rices, namely, cyanidin-3-O-glucoside (C3G) and peonidin-3-O-glucoside (P3G). The total anthocyanin content among pigmented genotypes ranged from 29.8 to 275.8 mg.100g^–1^ DW. Total phenolics ranged from 66.5 to 700.3 mg GAE.100g^–1^ DW with radical scavenging activity (RSA) varying between 17.7 and 65.7%. Anthocyanins and phenolics showed a direct relationship with RSA implying the nutraceutical benefits of deep pigmented rice such as Manipur black rice. Aromatic rices from Manipur were found to be genetically diverse. Therefore, efforts need to be made for maintaining the geographic identity of these rice and utilization in breeding for region-specific cultivar improvement.

## Introduction

Aromatic rice is superior-quality rice having fragrance along with other grain and cooking quality characteristics. Owing to these properties, they are popular among the consumers realizing a higher market value. In the rice gene pool, aromatic rice cultivars form a distinct group (Group V) as revealed by the isozyme analysis (Glaszmann, 1987; Khush, 2000). At the global level, most popular aromatic rices include Basmati rice from the Indo-Gangetic plains of the Indian subcontinent, Jasmine rice from Thailand, and Sadri rice from Iran.

North-eastern India is one of the major agro-biodiversity hotspots in the world, enriched with more than 10,000 diverse indigenous diverse rice cultivars including both aromatic and non-aromatic rice (Mao et al., 2009). Special among these are distinctly scented landraces such as *Joha* cultivars of Assam (Talukdar et al., 2017), *Chakhao* cultivars of Manipur, *Tai* cultivars of Mizoram, and *Kampti* cultivars of Arunachal Pradesh, which are grown and conserved by farmers over ages and distributed across different ecological niches (Durai et al., 2015; Roy et al., 2015). The Manipur state of North-eastern India is an isolated hilly region encircled by nine hill ranges and a central valley having climate varying from tropical to subalpine (GoM, 2018). *Chakhao*, meaning “delicious rice” in *Manipuri*, is the most popular aromatic rice of Manipur which also includes several lesser-known landraces. *Chakhao* cultivars have either pigmented (black, *amubi*) or non-pigmented (white, *angouba*) rice kernels. The cultivars with colored pericarp are distinct from other rice varieties originating from different parts of India (Gayacharan et al., 2018; Tulsiram et al., 2018). Particularly for sociocultural uses, farmers grow several of the *Chakhao* landraces such as *Chakhao Poireiton*, *Chakhao Amubi*, *Chakhao Sempak*, *Ching Chakhao*, *Chakhao Angouba* in local farm holds in smaller areas, covering less than 10% of the holdings. Historical accounts describe that black rice was restricted to the Royals, and the local *Meitei* community used it only during religious festivals and special occasions (Borah et al., 2018). Among the *Chakhao* rice, those with deep-pigmented kernels are popularly called “Manipur black rice.” They possess a high anthocyanin content in the pericarp, conferring antioxidant properties. Recognizing their exquisite nutraceutical quality, geographical indication (GI) status has been conferred to Manipur black rice in 2019 by the Government of India, registering it under GI No. 602 in the Geographical Indication Registry^1^ (GoI, 2019).

There are several landraces of aromatic rice of Manipur that share the common epithet *Chakhao* but remain seldom characterized. Earlier explorations during different periods conducted in Manipur have collected several such landraces that are conserved in the National Gene Bank (NGB) at the ICAR-National Bureau of Plant Genetic Resources (ICAR-NBPGR), New Delhi (Hore, 2005). In one study, the genetic diversity of 37 *Chakhao* landraces was assessed using 40 microsatellite (SSR) markers to reveal significant gene diversity (0.673) with markers having a PIC value of 0.63. These landraces were found grouped into six classes, having close correlation with farmers’ classification (Roy et al., 2014). Significant variation was also reported for yield-related traits in ten black rice genotypes of Manipur (Asem et al., 2019). By biochemical analyses, Asem et al. (2015) showed that the major anthocyanin fraction of black rice genotypes, *Chakhao Poireiton* and *Chakhao Amubi*, was delphinidin 3-galactoside, with *Chakhao Poireiton* having an average anthocyanin content of 740 mg/kg, and total phenolic content ranging from 5 to 6 g/kg of dried flour. Later, the 26 aromatic compounds were reported from *Chakhao Poireiton*, while 11 were reported from *Chakhao Amubi* (Asem et al., 2017). Another study by Chanu et al. (2016) reaffirmed the presence of high levels of anthocyanins, polyphenols, and zinc content having significant antioxidant activity in *Chakhao* landraces. However, the earlier studies suffered from one or other shortcomings, either having been carried out on a limited number of genotypes or having been characterized only for morphological, biochemical, or molecular variation. Therefore, a comprehensive study was felt necessary to assess the variation among several of the *Chakhao* rice including representative landraces, for agro-morphological, biochemical, and molecular diversity. There is no report of anthocyanin profiling and their variation across different black scented landraces cultivated in Manipur. Accordingly, the present study characterizes one of the comprehensive germplasm sets of aromatic rice landraces originating from Manipur including black rice, for phytochemical properties such as pigmentation, anthocyanin content, and antioxidant activities together with agro-morphological, molecular, and grain qualities such as cooking and aroma.

## Materials and Methods

**FIGURE 1:**
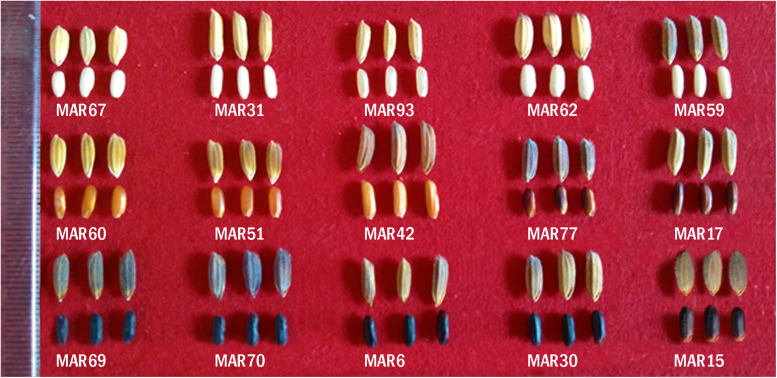
Variation for grain characters in a representative set of aromatic rice germplasm from Manipur. *Chakhao Angouba* (MAR 67), *Napnang Hangmei* (MAR 31), *Kabo Chakhao* (MAR 93), *Buhman* (MAR 62), *Buhman* (MAR 59), *Buhman* (MAR 60), *Buhman Te* (MAR 51), *Chakhao Phou* (MAR 42), *Ethe Buw* (MAR 77), *Chakhao* (MAR 17), *Chakhao Amubi* (MAR 69), *Chakhao Poireiton* (MAR 70), *Chakhao Poireitol* (MAR 6), *Chakhao Poireiton* (MAR 30), *Chakhao* (MAR 15). Note that genotypes with the same names have significant variation in grain types.

A total of 93 aromatic rice germplasm accessions collected from different parts of Manipur covering both hill and valley ecosystems were used in the study (Supplementary Table S1). Among these, 79 genotypes were sourced from NGB, ICAR-NBPGR, New Delhi; seven were collected from farmers’ field in Manipur and seven were sourced from ICAR-Regional Center for North-Eastern Hill Region (ICAR-RC-NEH), Manipur center. The genotypes were initially grouped based on *a priori* information on pericarp pigmentation, aroma, and adaptation ecologies (Table 1). The most contrasting feature of the study material was their diversity for spikelet and pericarp pigmentation (Figure 1). All the genotypes were initially multiplied at ICAR-Indian Agricultural Research Institute (ICAR-IARI), New Delhi, during *Kharif* 2017. During *Kharif* 2018, the genotypes were grown at the ICAR-RC-NEH Region, Manipur center, in lowland rainfed conditions. Each genotype was grown in three rows of 2.7 m length with a spacing of 20 cm between rows and 15 cm between plants. The field experiment was laid out in augmented design with four blocks and five non-aromatic checks, *viz*., RC Maniphou 7, RC Maniphou 10, RC Maniphou 11, RC Maniphou 12, and RC Maniphou 13. The experimental crop was raised with standard agronomic practices to maintain and harvest a good crop. The postharvest grain quality analysis, estimation of anthocyanin compounds, and molecular work were carried out at the Division of Genetics, and Division of Agricultural Chemicals, ICAR-IARI, New Delhi.

**TABLE 1:** Distribution of aromatic genotypes based on the various locations from which they originated in Manipur, included in the panel of genotypes used in the study based on *a priori* information such as pericarp color, aroma, ecosystem, and local names.

Attributes	Classes	Collection ecosystem		
		Hill	Valley	Unknown
Pericarp color	White	8	23	4
	Light brown	15	9	–
	Variegated brown	3	2	–
	Dark Purple	5	21	–
	Dark brown	–	1	–
	Variegated purple	–	2	–
Aroma	Strong	–	21	–
	Mild	22	31	4
	Undetected*	1	14	–
Local name	*Buhman* ^¶^	16	–	–
	*Chakhao*^¶^	6	57	4
	*Maklei*	4	–	–
	*Napnang Hangmei*	2	–	–
	*The Vumnu*	2	–	–
	*Ethe Buw*	1	–	–
	Black rice	–	1	–

*Those genotypes in which aroma could not be detected are included here. ^¶^Genotypes those share a common name in full or in part are grouped together.

### Agro-Morphological Characterization

Morphological observations were taken from five randomly selected uniform-looking plants within each line. Data was recorded on 12 quantitative traits including agro-morphological and grain quality traits and six qualitative pigmentation-related traits. The agro-morphological traits included days to fifty per cent flowering (DF), plant height (PH), panicle number (PN), panicle length (PL), 1000 grain weight (GW), and single plant yield (PY), while the grain quality traits included kernel length (KL), kernel breadth (KW), length–breadth ratio (LR), amylose content (AC), alkali spreading value (AS), and gel consistency (GC). Qualitative morphological information such as pigmentation status of basal leaf sheath (BL), ligule (LG), auricle (AU), and collar (CO) were recorded at the tillering stage as presence/absence, while the color of lemma/palea (LP) and pericarp (PC) was recorded at grain maturity on a scale of 1–9 as per the rice distinctness, uniformity, stability (DUS) guidelines (Rani et al., 2006). Grain quality traits such as amylose content (Juliano, 1971), alkali spreading value (Cagampang et al., 1973), and gel consistency (Little et al., 1958) were analyzed following standard evaluation system (SES) for rice (IRRI, 2013).

### Estimation of Anthocyanins, Total Phenols, and Antioxidant Activity in Grains

As anthocyanin is accumulated only in pigmented rice, the estimation of anthocyanin content was limited to a subset of thirty pigmented genotypes mostly with *Chakhao* nomenclature having either black, purple, or brown kernels with two cultivars with white rice kernels as non-pigmented checks. For the estimation of compounds, an anthocyanin-rich black rice extract (ABRE) was prepared from decorticated kernels using the method described by Sompong et al. (2011) with slight modification. Briefly, dehusked rice kernels were finely powdered in a mortar by manual grinding and stored at 4°C. About 100 mg of the flour was extracted with 25 ml of acidified methanol (HCl/methanol, 0.14% v/v) for 30 min at 40°C with ultrasonication two to three times to ensure complete color extraction. The extract was centrifuged at 8000 rpm for 5 min, and the supernatant was evaporated totally with a rotary evaporator (Heidolph Laborota 4001 efficient, Germany) at 40°C. The extract was reconstituted in 5 ml acidified distilled water (0.14% v/v concentrated HCl) and stored under refrigeration at −20°C till further analysis.

### Identification and Quantification of Anthocyanin Compounds

Identification of the anthocyanin compound in the black rice kernels was carried out with ABRE from *Chakhao Poireiton* (MAR70) by the liquid chromatography–mass spectrometry (LC-MS) system with Synapt G2 high-definition mass spectrometry (Waters Corp., Milford, Massachusetts) at the Advanced Instrumentation Research Facility, Jawaharlal Nehru University, New Delhi. The sample was eluted with water: methanol (90:10, v/v) with a flow rate of 0.1 ml/minute using the BEH C18 column of 2.1 × 100 mm with particle size 1.7 μm with temperature maintained at 25°C. Operating in a single quadrupole mode, LC-MS employed electrospray ionization (ESI). The instrument scanned over the mass (m)/charge (z) range of 100–1100 in the ESI positive ion mode (Lee, 2010).

Based on identified anthocyanin compounds in LC-MS, total anthocyanin content was quantified in different black rice genotypes by high-performance liquid chromatography (HPLC) as described by Lee (2010). The separation of anthocyanin compounds was carried out in reversed-phase separation with a C18 ODS Hypersil column (Thermo Electron Corporation; 250 × 4.6 mm, 5 μ). Chromatographic analysis was performed on the Waters^®^ HPLC system (Alliance 2695 separation module) with quaternary pumps, an autosampler, and a 2996 photodiode array (PDA) detector and driven by Empower 2 software for data recording.

Mobile phases composed of Solvent A containing water, acetonitrile, and trifluoroacetic acid (TFA) in the proportion 53:46:1 and Solvent B containing 0.1% TFA in HPLC-grade water (Singh et al., 2017) with a run time of 20 minutes. The gradient solvent system with Solvent A (20:60:20:20) and Solvent B (80:40:80:80) at 0–7 min, 7–11 min, 11–16 min, and 16–20 min, respectively, was used for maximum resolution. The flow rate was set at 600 μl per minute, and the column temperature was set at 25°C. The elution of the compounds was monitored at 517 nm wavelength, and peak pick was performed by comparing the retention time with the standard compound. The calibration curves were obtained for standard anthocyanin compounds by plotting different concentrations against the peak area in the chromatogram. By comparing the retention time and peak area with that of standard compounds, anthocyanin content in the sample was obtained.

### Quantification of Total Phenolics

To determine the total phenolic content in the ABRE, a modified Folin–Ciocalteu assay (Slinkard and Singleton, 1977; Saikia et al., 2012) was used. Briefly, an aqueous solution consisting of 100 μl of ABRE of the sample, 1.50 ml distilled water, and 100 μl of Folin–Ciocalteu reagent (2N) were mixed well. After 5 min., 300 μl of 20% sodium carbonate was added, mixed well, covered with silver foil, and kept at room temperature for 60 min. A blank was prepared similarly but by substituting the sample diluted mix with distilled water. The absorbance was measured at 765 nm using the Epoch 2 microplate reader (Biotech R, United States). A standard curve was prepared using different concentrations of gallic acid (100, 200, 300, 400, 500 μg.ml^–1^) from a stock solution of 10 mg.ml^–1^. The total phenolic content was calculated by the formula (CxV)/W, where C is the gallic acid equivalent (GAE) of the sample (mg.ml^–1^) obtained from the standard curve, V is the volume of the extract in ml, and W is the weight of the sample (g). Total phenolic content is expressed as mg GAE per 100g dry weight (DW).

### Antioxidant Activity

Antioxidant activity of the ABRE was tested using the 2,2-diphenyl-1-picrylhydrazyl (DPPH) radical scavenging activity (RSA) (Brand-Williams et al., 1995). Fresh DPPH solution (0.066 mM) was prepared by dissolving 0.0026 g in 100 ml of 95% methanol. 100 μl of the sample extract was added to 2.9 ml of freshly prepared DPPH solution and incubated in the dark at room temperature for 30 min. The absorbance was measured using a spectrophotometer at 517 nm against methanol as a blank and 100 μl of 0.1% acidified water in 2.9 ml of DPPH solution as a control. RSA was calculated and expressed in percentage as [(A_0_-A_s_)/A_0_] × 100, where A_0_ is the absorbance of control and A_s_ is the absorbance of the sample extract.

### Pigmentation of the Rice Kernels

*L*^∗^, *a*^∗^, and *b*^∗^ color scales were used to determine the pigmentation of decorticated grain samples using the Hunter-Lab Colorimeter system (Miniscan^®^ XE Plus 4500 L, Virginia, United States). The *L* value indicated the level of darkness (0–50) and lightness (51–100), the *a* scale of positive value indicated the redness and negative value the greenness, and the *b* scale indicated yellowness for the positive value and blueness for the negative value. All three values were required to completely describe an object’s color (Hunter and Harold, 1987) and color analysis was carried out as described by Murdifin et al. (2015).

### Characterization of Molecular Variation Using Microsatellite Markers

A panel of fifty SSR markers recommended by the generation challenge program (GCP) of the Consultative Group for International Agricultural Research (CGIAR) providing genome-wide coverage was used for analyzing the genetic diversity (Ali et al., 2011). Genotypes representing diverse groups of rice, namely, *indica* (IR64), tropical *japonica* (IRGC3764), temperate *japonica* (Taipei 309), *Aus* (Nagina 22), and aromatic (Taraori Basmati) were included as checks along with 93 germplasm accessions to assess their genetic relatedness and clustering. Leaf samples were collected from the individual genotype, and DNA was extracted using the cetyltrimethylammonium bromide (CTAB) method (Murray and Thompson, 1980). Amplification by polymerase chain reaction (PCR) was carried out with 25 ng template DNA, 5 pmol of each primer, and 2 × ready-to-use PCR master mix (Genei, Bangalore) in a 10 μl reaction mixture. The PCR amplification parameters included initial denaturation at 95°C for 5 min, followed by 35 cycles of thermal profile consisting of 95°C for 30 s, marker-specific annealing temperature for 30 s, and 72°C at 1 min, and a final extension at 72°C for 10 min. The amplicons were resolved on 3.5% metaphor agarose gel stained with ethidium bromide and visualized on GelDoc XR (Bio-Rad Laboratories Inc., United States). A ladder of 50 bp was used for comparison of allele size.

### Data Analyses

The quantitative data was tested for descriptive statistics and normality and analyzed for variance pattern, using STAR software (IRRI, 2014), running under R environment. Significant testing is carried out at a minimum probability level of 95%. The data were subjected to association analyses, and the uncorrelated variables were used for resolving morphological diversity using principal component analysis. Marker data were subjected to diversity analysis, using the simple matching coefficient (SMC) as the estimate of genetic distance (Sokal and Michener, 1958). The diversity matrix was subjected to clustering, using the unweighted neighbor joining method with bootstrapping 10,000 times. The diversity pattern was further resolved for population structure (Pritchard et al., 2000) using Structure v.2.3.4 (Prichard et al., 2010), and subpopulation genetic statistics were worked out using GenAlex v.6.5 (Peakall and Smouse, 2012).

## Results

### Variability in Agro-Morphological Traits

**FIGURE 2:**
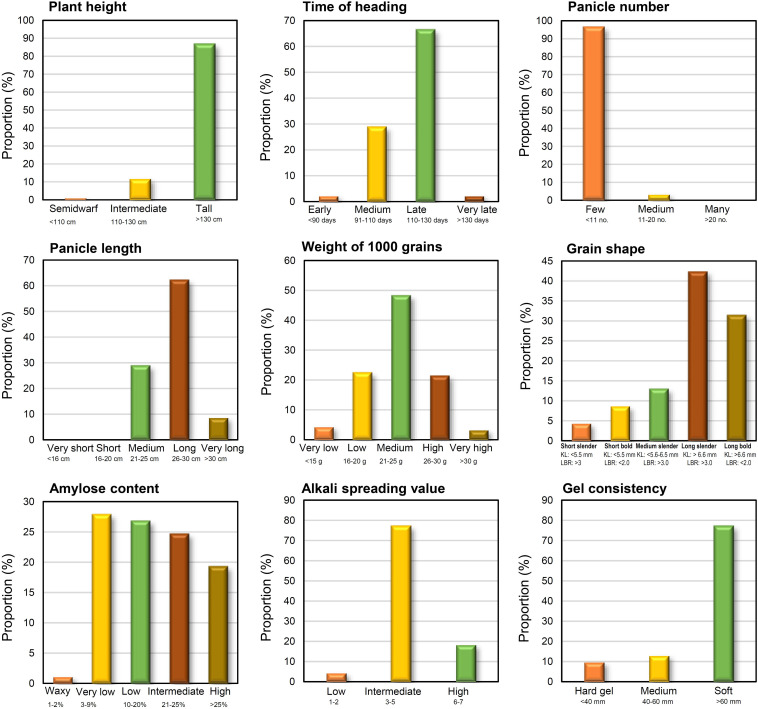
Distribution of aromatic rice genotypes of Manipur, based on agro-morphology and grain quality by standard evaluation system (SES) classes.

**FIGURE 3:**
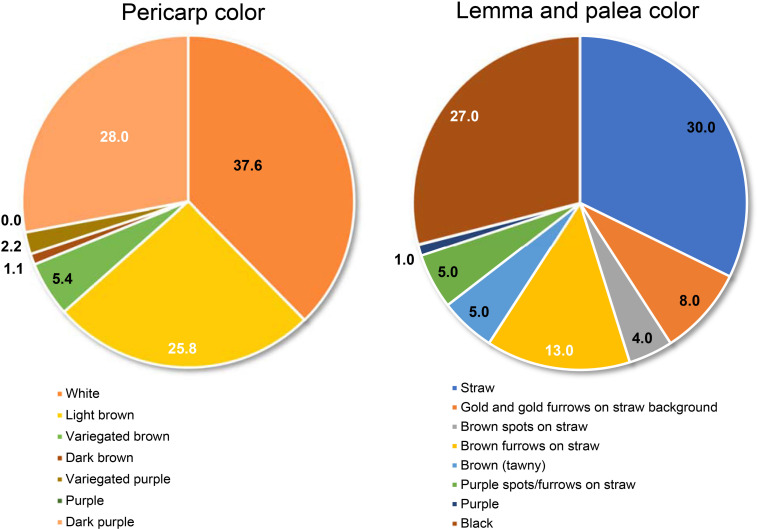
Distribution of genotypes based on the phenotypic variation for grain coloration in the aromatic rice germplasm of Manipur.

Trait-based frequency analysis of the aromatic rice germplasm is represented in Figure 2. Majority of these genotypes (66.7%) were late flowering (DFF: 111–130 days), tall (>130 cm) with a low number (<11) of longer panicles (26–30 cm), and having a medium range (21–25 g) of 1,000 grain weight. Observations on pigmentation on different parts of rice plants (Figure 3) revealed that 42 genotypes (45.2%) showed a purple pigmentation of the basal leaf, 36 genotypes (38.7%) possessed a purple ligule, and 44 genotypes (47.3%) produced a purple auricle and collar. Straw-colored lemma and palea were predominant followed by a black color. Grain quality assessment revealed that majority of them were long and slender followed by a long bold category. Further, most of the genotypes had low amylose content combined with soft gel consistency and intermediate alkali spreading value. 26% of the genotypes were found to possess a dark purple pericarp.

**FIGURE 4:**
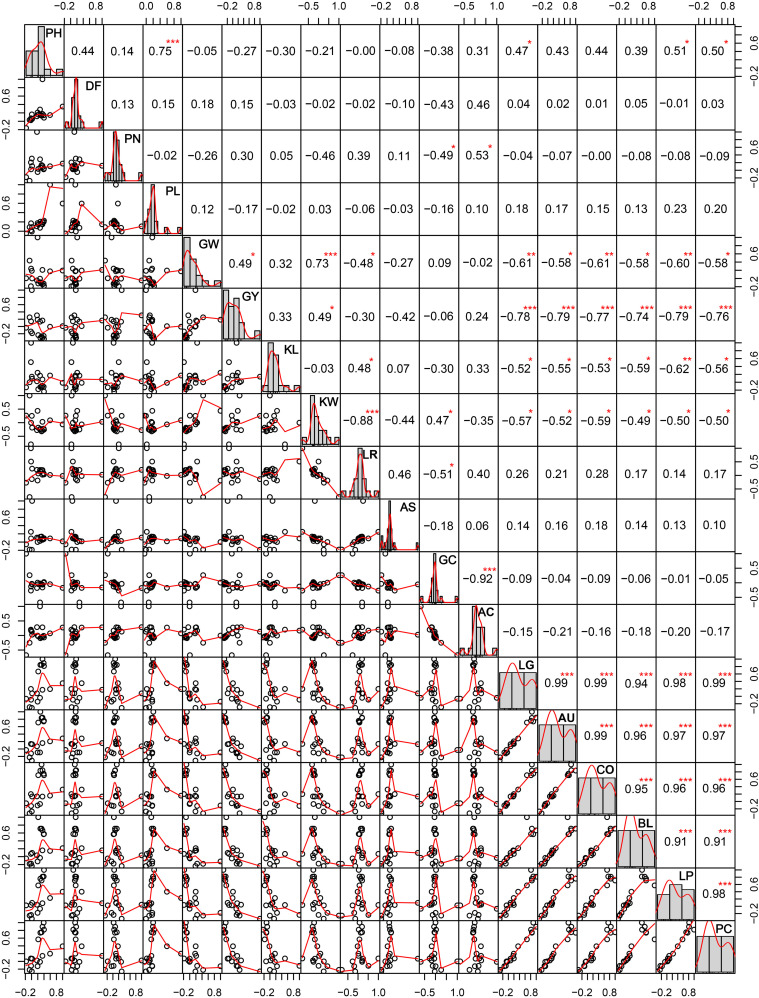
Correlations among different agro-morphological traits in the aromatic rice germplasm of Manipur. PH, plant height in cm; DF, days to 50% flowering; PN, panicle number; PL, panicle length in cm; GW, weight of 1,000 grains in g; GY, grain yield per plant in g; KL, kernel length in mm; KW, kernel width in mm; LR, length to width ratio; AS, alkali spreading value; GC, gel consistency; AC, apparent amylose content in percentage; LG, ligule pigmentation; AU, auricle pigmentation; CO, collar pigmentation; BL, basal leaf color; LP, lemma and palea color; PC, pericarp color. *, **, *** Significant at probability levels of 0.05, 0.01 and 0.001, respectively.

The correlation among different agro-morphological traits (Figure 4) depicted that PH and PL were significantly positively related and KW significantly influenced GW and GY. Among the cooking quality traits, GC was negatively correlated with AC. All the pigmentation parameters were negatively associated with GW and GY. It was observed that pigmentation of vegetative plant parts (BL, LG, AU, and CO) was significantly correlated with seed pigmentation (LP and PC).

Analysis of variance (ANOVA) for different agro-morphological traits was assessed (Table 2) between pigmented (27) versus non-purple color groups (71) and based on the adaptation ecology and valley (58) versus hill (31) regions. A significant difference was observed between the two-color groups for traits like PH, PL, and GY. The deep purple genotypes were taller and produced longer panicles and lower grain yield as compared to non-deep purple genotypes. Among regions, valley genotypes were taller and possessed a longer panicle length as compared to hill genotypes.

**TABLE 2:** Analysis of variance for different agro-morphological and grain quality traits in the aromatic rice germplasm of Manipur over *a priori* classification based on pericarp color and ecosystem adaptation.

Traits	Pericarp color class					Ecosystem class				
	Category	Count	Mean	CV%	*p-*value	Category	Count	Mean	CV%	*p-*value
PH	LB	24	145.5	15.2	0.00	Valley	58	162.6	8.1	0.00
	White	35	154.0	12.0		Hill	31	143.7	13.2	
	DP/VP/DB	34	163.2	6.9						
DF	LB	24	107.7	10.1	0.00	Valley	58	115.1	8.7	0.07
	White	35	116.5	9.2		Hill	31	111.0	9.6	
	DP/VP/DB	34	116.4	7.0						
PN	LB	24	7.2	36.6	0.08	Valley	58	7.3	23.7	0.52
	White	35	6.9	30.7		Hill	31	7.0	36.8	
	DP/VP/DB	34	7.4	20.5						
PL	LB	24	25.5	8.5	0.08	Valley		27.0	8.5	0.03
	White	35	26.3	10.4		Hill		25.8	9.8	
	DP/VP/DB	34	26.9	7.8						
GW	LB	24	23.9	22.6	0.08	Valley	58	25.2	21.6	0.07
	White	35	25.6	21.8		Hill	31	22.9	26.9	
	DP/VP/DB	34	22.9	19.3						
GY	LB	24	12.8	78.5	0.01	Valley	58	11.6	68.5	0.54
	White	35	14.6	54.6		Hill	31	12.7	59.3	
	DP/VP/DB	34	8.9	62.8						
KL	LB	24	6.3	7.8	0.32	Valley	58	6.2	6.9	0.59
	White	35	6.3	8.1		Hill	31	6.3	8.5	
	DP/VP/DB	34	6.1	6.1						
KW	LB	24	2.2	8.2	0.01	Valley	58	2.2	13.1	0.22
	White	35	2.2	13.8		Hill	31	2.1	11.1	
	DP/VP/DB	34	2.1	10.8						
LR	LB	24	2.9	13.3	0.22	Valley	58	2.9	14.2	0.16
	White	35	2.9	13.9		Hill	31	3.0	11.9	
	DP/VP/DB	34	3.0	12.9						
AS	LB	24	2.9	38.4	0.30	Valley	58	5.0	19.3	0.29
	White	35	2.9	41.6		Hill	31	4.8	19.2	
	DP/VP/DB	34	3.0	28.2						
GC	LB	24	113.7	36.6	0.08	Valley	58	91.5	46.2	0.01
	White	35	91.4	42.9		Hill	31	116.2	29.9	
	DP/VP/DB	34	92.7	44.1						
AC	LB	24	14.0	64.7	0.06	Valley	58	17.7	51.7	0.07
	White	35	19.5	45.2		Hill	31	14.2	57.7	
	DP/VP/DB	34	16.6	51.1						

*PH, plant height in cm; DF, days to 50% flowering; PN, panicle number; PL, panicle length in cm; GW, weight of 1,000 grains in g; GY, grain yield per plant in g; KL, kernel length in mm; KW, kernel width in mm; LR, length-to-width ratio; AS, alkali spreading value; GC, gel consistency; AC, apparent amylose content in percentage; LB, light brown; DP, dark purple; VP, variegated purple; DB, dark brown.*

**FIGURE 5:**
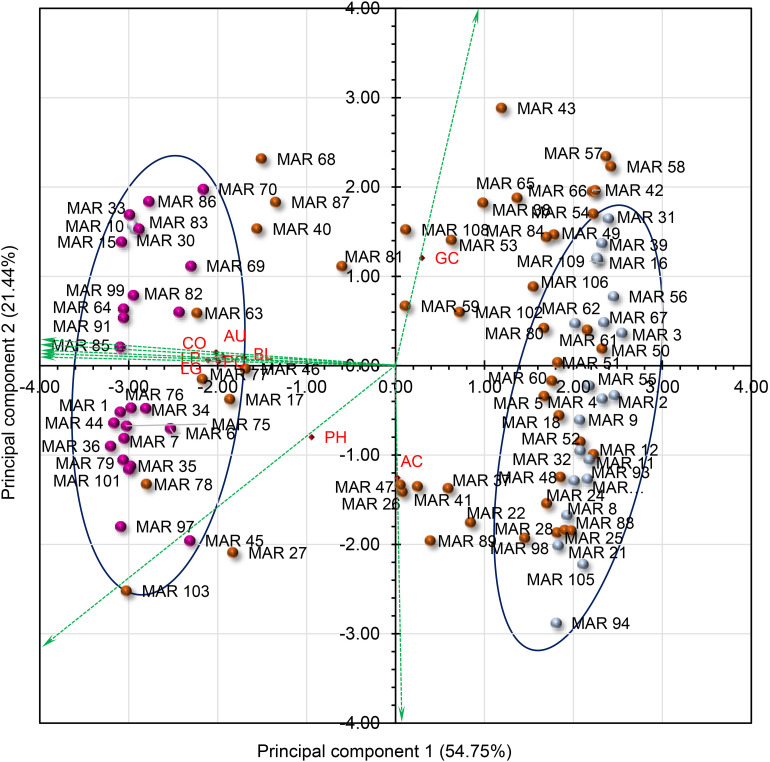
PCA biplot showing the partitioning of Manipur aromatic germplasm using most variable phenotypic traits. There were three groups of genotypes, grouped based on pericarp color. The genotypes with white pericarp (light blue) formed the cluster 1, while deep purple-colored (purple) genotypes formed the second. The third group was intermediate to the first two (brown). The green arrows show the direction of influence of the traits. LG, ligule pigmentation; AU, auricle pigmentation; CO, collar pigmentation; BL, basal leaf color; LP, lemma and palea color; PC, pericarp color; PH, plant height in cm; GC, gel consistency; AC, amylose content.

Based on principal component analysis (PCA) using all the agronomic, pigmentation, and grain quality traits, the seven most variable phenotypic traits were identified contributing significantly to the total phenotypic variation (Supplementary Table S2). Using the subset traits, the first two principal components (PC) accounted for 76% of the total variance (Table 3) with the first PC accounting for 55% of the total variation. The main contributing variables to the first PC were pigmentation status of plant organs, namely, LG, CO, AU, PC, LP, and BL. The second principal component contributed 21% of the total variation and had influence from AC, GC, and PH. The PCA biplot (Figure 5) dispersed genotypes clearly across PC1 and PC2. Most of the deep purple and white pericarp genotypes were clustered separately in opposite directions along the PC1 axis, whereas other color categories such as light brown, dark brown, variegated purple, and variegated brown were found dispersed in between. Similarly, the tall genotype MAR103 (*Chakhao Amubi*) (177.4 cm) and dwarf genotype MAR43 (*Chakhao Phou*) (98.0 cm) were placed diagonally opposite along the PC2 axis. Genotypes with low amylose were found coupled with high GC values (MAR43, MAR57, MAR58), which were clearly separated from genotypes with high amylose and low GC values (MAR94, MAR105).

**TABLE 3:** Principal components extracted from the most influential phenotypic traits among the aromatic rice germplasm of Manipur.

Components	EV	Var	Contribution of traits (%)								
			LG	AU	CO	BL	LP	PC	PH	GC	AM
PC1	4.93	0.55	18.3	16.8	16.8	12.0	15.6	16.4	3.7	0.4	0.0
PC2	1.93	0.21	0.1	0.6	0.2	0.2	0.3	0.1	17.1	39.2	42.2
PC3	0.68	0.08	0.2	1.4	0.7	19.6	2.8	2.6	56.7	13.3	2.7

*EV, eigenvalue; Var, proportion of total variation; LG, ligule pigmentation; AU, auricle pigmentation; CO, collar pigmentation; BL, basal leaf color; LP, lemma and palea color; PC, pericarp color; PH, plant height in cm; GC, gel consistency; AM, amylose content.*

### Molecular Variation Based on SSR Markers

Among the fifty SSR markers used, two markers (RM 133 and RM 484) were found to be monomorphic across the germplasm and hence excluded from further analysis. The summary statistics of 48 SSR markers is presented in Table 4. A total of 171 alleles were identified, with an average of 3.5 alleles per marker while the number of alleles per marker varied from 2 to 7. A maximum number of seven alleles each were detected with RM 413, RM 552, and RM 144. The major allele frequency was lowest for RM 552 (0.206) and highest for RM 454 (0.959) with a mean of 0.664 (Supplementary Table S3). The gene diversity or expected heterozygosity ranged from 0.115 (RM125) to 0.826 (RM 552) with a mean value of 0.443. The chromosome level diversity was maximum for chromosome 11 (0.624), while the minimum diversity was observed in chromosome 6 (0.281). The highest PIC value was obtained for RM 552 (0.802) and lowest for RM 454 (0.078) with a mean of 0.394. Seventeen rare alleles with frequencies less than 5% across accessions were identified in this study. Further, there were 11 unique alleles also. Three of these unique alleles were found in genotypes MAR50, MAR51, and MAR58, all of which had *Buhman* as part of its name.

**TABLE 4:** Marker polymorphism and allelic status of GCP panel microsatellite markers among aromatic rice germplasm from Manipur.

Chr	No. of SSR markers	Frequency of multiallelic loci						PIC	
		2	3	4	5	6	7	Mean	Range
Chr 1	8	2	2	3	1	–	–	0.358	0.179–0.447
Chr 2	2	–	1	1	–		–	0.502	0.403–0.600
Chr 3	5	1	4	–	–		–	0.299	0.204–0.383
Chr 4	2	1	–	–	–	1	–	0.430	0.166–0.694
Chr 5	5	1	2	–	–	1	1	0.380	0.157–0.741
Chr 6	3	1	2	–	–	–	–	0.250	0.075–0.386
Chr 7	4	3	–	–	1	–	–	0.258	0.108–0.419
Chr 8	7	2	1	3	1	–	–	0.419	0.182–0.591
Chr 9	3	–	2	1	–	–	–	0.417	0.364–0.498
Chr 10	3	–	1	1	–	1	–	0.488	0.282–0.599
Chr 11	4	–	1	1	–	–	2	0.587	0.233–0.802
Chr 12	2	–	1	1	–	–	–	0.506	0.455–0.556
Overall	48	11	17	11	3	3	3	0.394	0.075–0.802

*Chr, chromosome; PIC, polymorphism information content.*

### Cluster Analysis Using Molecular Data

**FIGURE 6:**
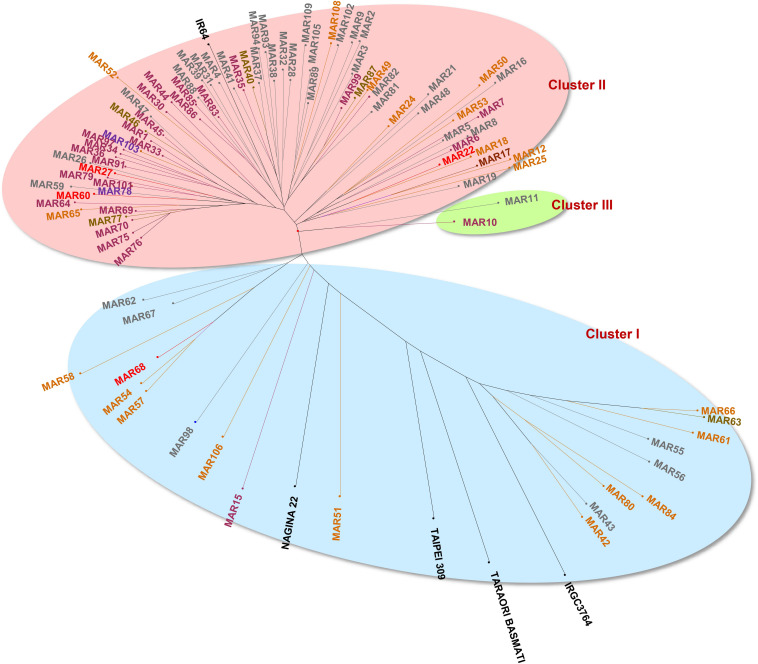
Hierarchical clustering of aromatic rice genotypes from Manipur based on SSR markers by unweighted neighbor joining using simple matching coefficients showing three distinct clusters.

The genetic distance estimated through SMC, between every pair of genotypes, varied between 0.08 and 0.86 with an average of 0.47. The SMC dissimilarity matrix across 98 genotypes, which included 93 aromatic rice germplasm and five check lines, were used to group the genotypes into three major clusters (Figure 6). Cluster I contained 23 genotypes distributed in three subgroups of 12, 5, and 6 genotypes included *japonica*, *Basmati*, and *aus* checks, respectively. Most of the members from Cluster I were from the hill region and possessed a light brown pericarp color. Cluster II included 73 genotypes mostly grown under valley ecology. It included subgroups of pigmented (deep purple, variegated brown, variegated purple), white and light brown pericarp genotypes. Cluster II also included the *indica* check, IR64. Cluster III contained only two *Chakhao* genotypes, MAR10 and MAR11, originating from the valley region.

### Population Structure of Aromatic Rice Accessions From Manipur

**FIGURE 7:**
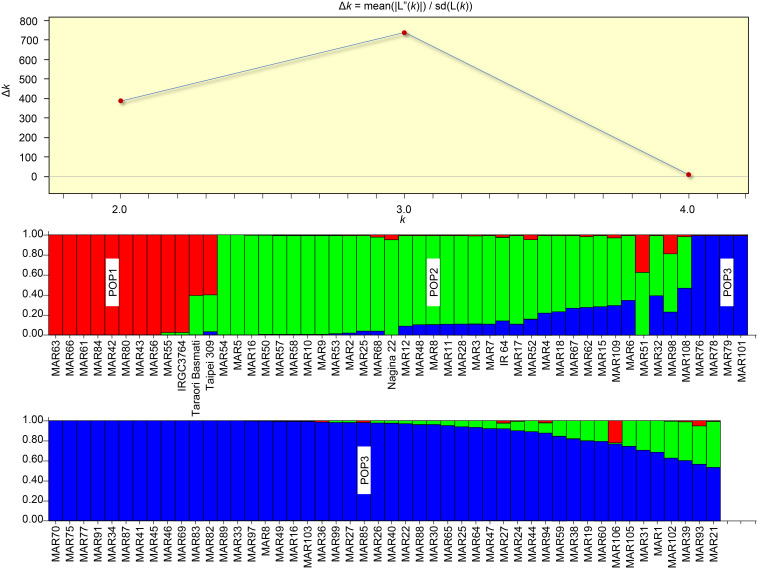
Population structure of aromatic rice germplasm from Manipur. Three subpopulations were resolved by the highest ΔK value (above). Among the subpopulations, POP1 showed more distinctness from other two. POP1 also was the group that did not have genetic admixing. POP2 and POP3 shared a large set of admixtures.

The Bayesian analysis of the population structure of aromatic rice of Manipur revealed three subpopulations as indicated by the *ad hoc* statistic, ΔK (Figure 7). The subpopulations, POP1, POP2, and POP3 included 12, 34, and 52 genotypes, respectively. It was interesting to note that the cluster I subgroups from the earlier analysis were bifurcated into two subpopulations (POP1 and POP2). The members of each subpopulation were further divided as pure or admixed based on inferred ancestry coefficients. Those genotypes with the coefficient of =0.95 were counted as pure types, and those with coefficients < 0.95 were counted as admixtures. Accordingly, POP1 contained nine pure genotypes, which included six of the hill accessions with white and light brown pericarp. The remaining members of this group included a landrace, “*Maklei*,” with variegated brown pericarp, the tropical *japonica* check, IRGC3764, and the temperate *japonica* check, Taipei 309. The aromatic group check, Taraori Basmati, was grouped along with *japonica* check, in POP1 as admixture. These hilly genotypes were also grouped as distinct by cluster analysis. They showed a distinctly discernible allelic pattern for the biallelic markers, namely, RM 489, RM 338, RM 161, RM 455, and RM 284, from other genotypes. POP2 included 12 genotypes as pure types and 22 genotypes as admixtures that included *indica* check, IR64, and the *aus* check, Nagina 22. These genotypes either belonged to hill and valley regions or were mostly with white and light brown pericarp. Two *Chakhao* genotypes, MAR10 and MAR11, which formed Cluster III were included in this subpopulation. POP3, the largest subpopulation, included 29 genotypes as pure and 23 genotypes as admixtures. Most of the genotypes (22) in the pure category were dark pigmented (deep purple, variegated purple/brown) from both valley and hill regions.

The analysis of molecular variance (AMOVA) revealed significant variation among and within subpopulations obtained (Table 5). Among subpopulations, a variance of 23% was found, however, within subpopulations 77% variance was obtained among individuals. No within-individual variation was found. The population-specific F_st_ of the three subpopulations were 0.260, 0.401, and 0.106, respectively, with an average of 0.256 indicating higher level of genetic differentiation.

**TABLE 5:** Analysis of molecular variance (AMOVA) and population differentiation statistics among the subpopulations of Manipur aromatic rice panel.

Source	df	SS	MS	Ve	PVE (%)
Among populations	2	359.5	179.8	2.79	23.0
Among individuals	95	1804.1	19.0	9.49	77.0
Within individuals	98	0.000	0.0	0.00	–
Total	195	2163.6		12.3	100.0

**Population statistics**	**F_st_**	**G_st_**	**G′_st_**	**G′_st_ (Hed)**	**D*_*est*_***

POP1–POP2	0.177	0.150	0.261	0.424	0.322
POP1–POP3	0.250	0.227	0.370	0.549	0.417
POP2–POP3	0.069	0.055	0.105	0.121	0.069

*df, degrees of freedom; SS, sum of squares; MS, mean square; Ve, estimated variance; PVE, percentage of total variation explained; F_*st*_, fixation statistic (Wright, 1951); G_*st*_, G statistic (Nei, 1973); G’_*st*_, standardized G_*st*_ (Nei, 1986); G′_*st*_ (Hed), Hedrick’s G_*st*_ (Hedrick, 2000); D_*est*_, Jost’s D (Jost, 2008).*

### Quantification of Anthocyanins, Polyphenols, and Antioxidant Activity

**FIGURE 8:**
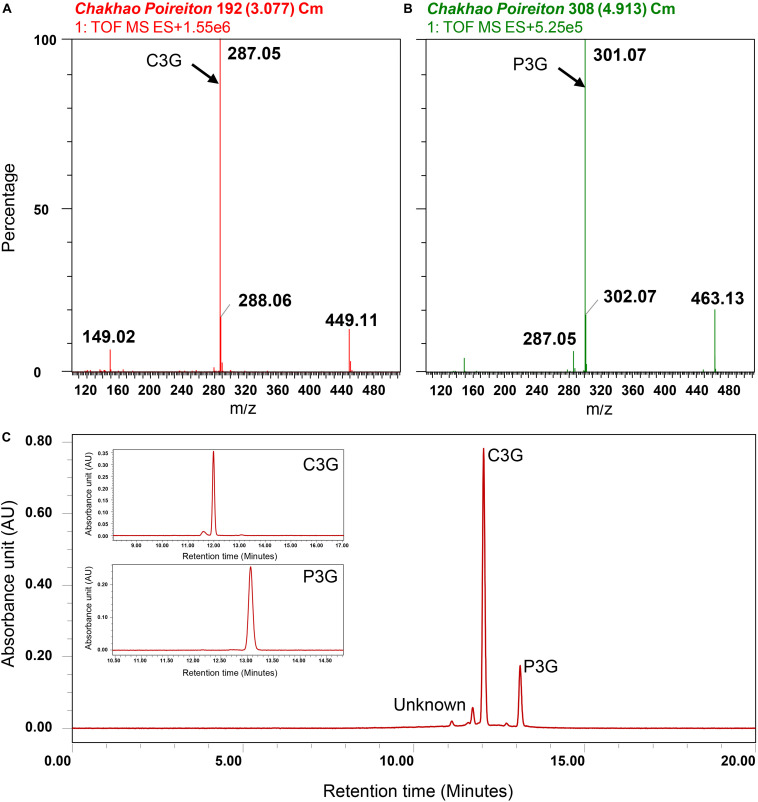
Identification of anthocyanins in Manipur black rice cultivar *Chakhao Poireiton.* MS spectra depicting **(A)** cyanidin-3-o-glucoside (C3G) fragments, **(B)** peonidin-3-o-glucoside (P3G) fragments. Quantification of identified anthocyanins by high-performance liquid chromatography **(C)** Chromatogram of *Chakhao Poireiton*, with standard C3G and P3G peaks as insets.

In the ARBE of *Chakhao poireiton* (MAR70), by mass spectrometry, two peaks were detected at m/z of 449.1 and 463.1 corresponding to C3G and P3G, respectively, together with two major peaks at 287.05 and 301.07 (Figure 8), which could be identified as cyanidin and peonidin, the aglycons. There were also minor peaks detected which could not be identified due to their very low concentration in the extract. They are quantified subsequently as cyanidin-3-glucoside equivalents (C3GE). The major anthocyanin fractions were further confirmed chromatographically by their retention time (RT) of 11.95 min for C3G and 12.9 min for P3G, as obtained from the corresponding standards (Supplementary Table S4). There were also minor peaks at lower RT, which are unidentified. Quantification of anthocyanins from thirty genotypes, identified C3G as the major anthocyanin fraction, were followed by P3G (Figure 8). There was a significant variation among black pigmented genotypes for total anthocyanins that ranged from 29.8 to 275.8 mg.100g^–1^ (Table 6). The genotypes with similar names like *Chakhao Poireiton*, *Chakhao Amubi*, and *Ching Chakhao* collected from different places showed significant variation for total anthocyanin content and its constituent compounds (Table 6). The average C3G content (113.3 mg.100g^–1^) among the genotypes was almost six times higher than the average P3G content (19.7 mg.100g^–1^) and seven times higher than C3GE (16.2 mg.100g^–1^). On average, the percent composition of C3G, P3G, and C3GE was 76, 13, and 11, respectively. Total phenolic content was found to be significantly different between white (56.0 mg GAE.100g^–1^ DW) and black (339.2 mg GAE.100g^–1^ DW) rice genotypes. RSA (%) by DPPH assay depicted a significant variation between white (7% on average) and pigmented genotypes assessed (38% on average). Interestingly, among the pigmented genotypes, minimum (17.7) and maximum (65.7) RSA (%) values were observed in deep purple genotypes, *Ching Chakhao Amubi* (MAR1) and *Chakhao* (MAR 91), respectively.

**TABLE 6:** Variation in total anthocyanins, total phenolics, radical scavenging activity, and color scales in the kernels of thirty pigmented rice landraces in comparison with two white rice genotypes from Manipur.

Code	Cultivar name	TA	C3G	P3G	C3GE	TP	RSA	L*	a*	b*
MAR 21	*Kabo Chakhao*	0.0^a^	0.0^a^	0.0^a^	0.0^a^	50.6 ± 1.9^a^	6.0	33.9 ± 2.1^o^	0.5 ± 0.2^a–d^	8.9 ± 1.1^j^
MAR 31	*Chakhao Angouba*	0.0^a^	0.0^a^	0.0^a^	0.0^a^	61.3 ± 0.8^b^	8.0	48.5 ± 1.0^p^	0.14 ± 0.1^a^	9.8 ± 0.5^j^
MAR 17	*Chakhao*	29.8 ± 1.9^b^	16.2 ± 0.3^b^	2.9 ± 0.1^b^	11.4 ± 0.3^cd^	156.3 ± 1.9^h^	20.7	20.7 ± 0.9^kl^	4 ± 0.6^k^	5.4 ± 0.7^g^
MAR 78	*Black rice*	36.0 ± 1.4^b^	22.2 ± 1.0^c^	3.3 ± 0.2^bc^	10.4 ± 0.2^c^	66.5 ± 1.5^c^	22.2	23.7 ± 0.8^n^	6.2 ± 0.7^m^	7.5 ± 0.8^i^
MAR 46	*Chakhao Amubi*	44.4 ± 1.7^c^	28.9 ± 1.4^d^	4.6 ± 0.2^bcd^	10.9 ± 0.4^cd^	74.6 ± 2.5^d^	19.0	21.5 ± 0.5^lm^	3.0 ± 0.8^j^	4.8 ± 0.9^g^
MAR 77	*Ethe Buw*	45.2 ± 4.0^c^	29.1 ± 1.5^d^	5.3 ± 0.3^cde^	10.7 ± 0.2^cd^	156.5 ± 2.5^h^	19.9	21.8 ± 0.8^lm^	5.3 ± 0.4^l^	4.8 ± 0.7^g^
MAR 83	*Langphou Chakhao*	45.2 ± 3.1^c^	29.5 ± 1.9^d^	3.8 ± 1.0^bc^	11.8 ± 0.3^cde^	130.4 ± 2.6^f^	23.5	19.6 ± 0.8^jk^	4.6 ± 0.7^k^	2.7 ± 0.6^def^
MAR 1	*Ching Chakhao Amubi*	57.9 ± 1.3^d^	39.8 ± 1.0^e^	5.6 ± 0.2^cde^	12.5 ± 0.1^def^	83.4 ± 2.6^e^	17.7	22.5 ± 0.6^m^	4.0 ± 0.8^k^	6.3 ± 0.4^h^
MAR 15	*Chakhao*	65.2 ± 3.6^e^	46.3 ± 2.2^f^	7.6 ± 0.7^ef^	11.2 ± 1.0^cd^	146.7 ± 1.7^g^	36.6	19.5 ± 0.3^jk^	2.1 ± 0.1^i^	2.9 ± 0.1^def^
MAR 76	*The Vumnu*	74.1 ± 0.4^f^	54.1 ± 0.4^g^	6.3 ± 0.5^def^	13.7 ± 0.2^e–h^	207.4 ± 2.6^j^	24.2	16.1 ± 0.6^a–e^	0.6 ± 0.1^a–d^	1.3 ± 0.6^c^
MAR 99	*Chakahao Huikap*	81.4 ± 3.4^g^	55.9 ± 2.4^g^	11.6 ± 1.2^g^	13.9 ± 0.4^fgh^	306.9 ± 4.2^n^	30.7	16.9 ± 0.4^b–e^	4.1 ± 0.4^k^	2.2 ± 0.0^d^
MAR 40	*Chakhao Amubi*	82.2 ± 3.8^g^	50.7 ± 4.6^*f*g^	17.9 ± 0.8^h^	13.5 ± 1.7^efg^	220.7 ± 1.2^l^	25.6	20.2 ± 0.3^k^	2.3 ± 0.2^i^	3.2 ± 0.2^ef^
MAR 75	*The Vumnu*	82.9 ± 0.3^g^	54.4 ± 0.7^g^	13.2 ± 0.4^g^	15.3 ± 0.1^ghi^	297.4 ± 2.7^m^	40.9	16.1 ± 0.6^a–d^	1.2 ± 0.5^d–h^	0.3 ± 0.6^b^
MAR 79	*Buhman*	88.5 ± 2.5^g^	66.3 ± 2.0^h^	8.6 ± 0.5^f^	13.5 ± 0.3^efg^	175.8 ± 2.5^i^	30.6	15.9 ± 0.5^abc^	1.8 ± 0.2^g–i^	−0.2^ab^
MAR 34	*Chakhao Amubi*	133.4 ± 3.6^h^	112.2 ± 2.0^jk^	13.8 ± 1.6^g^	7.3 ± 0.3^b^	327.4 ± 4.4^p^	26.2	18.7 ± 0.5^ij^	0.8 ± 0.1^a–d^	2.5 ± 0.7^de^
MAR 101	*Chakhao*	142.7 ± 3.5^i^	103.2 ± 2.4^i^	20.3 ± 1.2^ij^	19.3 ± 0.7^j^	495.8 ± 7.3^w^	35.9	15.2 ± 0.7^a^	1.8 ± 0.2^f–i^	0.0 ± 0.1^ab^
MAR 82	*Chakhao Amubi*	145.2 ± 7.6^i^	111 ± 6.4^j^	19.4 ± 1.7^hi^	14.7 ± 0.5^gh^	384.9 ± 2.7^r^	39.8	15.9 ± 0.4^ab^	0.7 ± 0.4^a–d^	−0.3^ab^
MAR 45	*Chakhao*	146.3 ± 0.8^i^	110 ± 3.9^j^	22.7 ± 2.5^k^	13.5 ± 0.7^efg^	326.7 ± 1.9^p^	36.2	18.4 ± 0.4^g–j^	1.7 ± 0.4^e–i^	2.5 ± 0.3^de^
MAR 7	*Chakhao Poireitol*	157.9 ± 1.3^j^	118.8 ± 1.3^l^	26.5 ± 0.1^lm^	12.5 ± 0.1^def^	213.6 ± 4.9^k^	42.6	17.2 ± 0.3^b–g^	1.3 ± 0.1^c–g^	2.9 ± 0.3^def^
MAR 30	*Chakhao Poireiton*	159.7 ± 5.4^j^	116.6 ± 3.0^kl^	22.3 ± 0.9^jk^	20.7 ± 3.5^j^	297.6 ± 4.1^m^	37.7	18.7 ± 0.7^hij^	1.3 ± 0.3^d–h^	2.7 ± 0.3^def^
MAR 35	*Chakhao Poireiton*	184.6 ± 6.0^k^	158.6 ± 2.2^n^	17.4 ± 3.2^h^	8.5 ± 2.3^b^	315.0 ± 1.6^o^	32.9	18.7 ± 0.6^ij^	1.1 ± 0.03^c–g^	2.4 ± 0.4^de^
MAR 103	*Chakhao Amubi*	197.3 ± 2.9^l^	149.5 ± 2.7^lm^	25.3 ± 0.6^l^	22.4 ± 1.0^k^	504.6 ± 3.2^x^	47.1	23.8 ± 0.6^n^	4.2 ± 0.6^k^	5.5 ± 0.3^gh^
MAR 64	*Maklei*	207.1 ± 3.1^m^	161.6 ± 2.8^n^	28.5 ± 0.7^m^	16.9 ± 0.6^i^	447.5 ± 2.7^u^	50.0	17.3 ± 0.7^c–h^	1.2 ± 0.4^d–g^	−0.04^ab^
MAR 70	*Chakhao Poireiton*	230.9 ± 6.9^n^	183.1 ± 5.0^o^	27.9 ± 1.8^m^	19.7 ± 2.0^j^	589.2 ± 1.6^z^	48.4	18.2 ± 0.5^f–j^	0.5 ± 0.3^a–d^	−0.5^ab^
MAR 69	*Chakhao Amubi*	231.9 ± 3.6^n^	184.4 ± 0.5^o^	27.7 ± 2.5^m^	19.4 ± 1.2^j^	525.1 ± 3.4^y^	38.1	17.0 ± 0.8^b–f^	0.7 ± 0.2^a–d^	−0.5^ab^
MAR 36	*Chakhao Pungdol Angouba*	234.4 ± 9.4^n^	193.7 ± 7.5^p^	25.1 ± 1.6^l^	15.5 ± 0.5^hi^	418.5 ± 0.9^s^	51.5	18.7 ± 0.6^ij^	1.2 ± 0.1^c–g^	2.4 ± 0.3^de^
MAR 44	*Chakhao Poireiton*	243.7 ± 7.0^o^	192 ± 4.5^p^	24.5 ± 2.6^kl^	27.1 ± 0.2^l^	443.0 ± 3.8^t^	49.7	18.7 ± 0.4^g–j^	0.8 ± 0.1^a–d^	2.4 ± 0.3^de^
MAR 33	*Chakhao Poireiton*	259.2 ± 4.1^p^	191.8 ± 4.8^p^	56.3 ± 2.1^p^	11.0 ± 1.3^cd^	672.3 ± 2.4^b^	57.7	18.2 ± 0.4^f–j^	1.0 ± 0.1^b–e^	2.5 ± 0.1^def^
MAR 85	*Chakhao*	260.8 ± 2.2^p^	204.1 ± 1.0^q^	33.1 ± 1.4^n^	23.5 ± 0.5^k^	461.3 ± 2.7^v^	46.3	17.4 ± 0.5^d–i^	0.7 ± 0.1^a–d^	−0.7^a^
MAR 6	*Chakhao Poireitol*	264.0 ± 5.2^pq^	204.2 ± 4.6^q^	33.5 ± 1.1^n^	26.2 ± 0.5^l^	364.5 ± 3.0^q^	59.3	17.4 ± 0.7^d–i^	1.9 ± 0.8^hi^	3.5 ± 0.6^f^
MAR 86	*Ching Chakhao*	269.4 ± 4.4^qr^	202 ± 5.3^q^	32.0 ± 0.2^n^	35.3 ± 1.1^m^	667.1 ± 3.2^a^	52.9	15.3 ± 0.9^a^	0.2 ± 0.1^ab^	−0.2^ab^
MAR 91	*Chakhao*	275.8 ± 5.1^r^	207.6 ± 3.6^q^	44.7 ± 1.2^o^	23.5 ± 0.7^k^	700.3 ± 2.8^c^	65.7	17.5 ± 0.4^e–i^	0.4 ± 0.1^abc^	−0.16^ab^

TA, total anthocyanin in mg.100g^–1^ dry weight (DW); C3G, cyanidin-3-O-glucoside in mg.100g^–1^ DW; P3G, peonidin-3-O-glucoside mg.100g^–1^ DW; C3GE, cyanidin-3-glucoside equivalents in mg.100g^–1^ DW; TP, total phenolics in mg gallic acid equivalent (GAE) 100g^–1^; RSA, radical scavenging activity in percentage; L*, a*, b* are CIELAB color scales. Means with different letter(s) indicate significant difference between genotypes at p = 0.05 by Duncan’s multiple-range test.

Apparently, color values *L*^∗^ and *b*^∗^ were higher in white genotypes as compared to deep black-pigmented genotypes. The *b*^∗^ value was negative in deeply pigmented genotypes. The parameter *a*^∗^ depicting redness–greenness was higher in low anthocyanin-containing genotypes (brown) as compared to white and dark purple pigmented genotypes.

### Relationship of Pigmentation Features and Nutraceutical Properties

**FIGURE 9:**
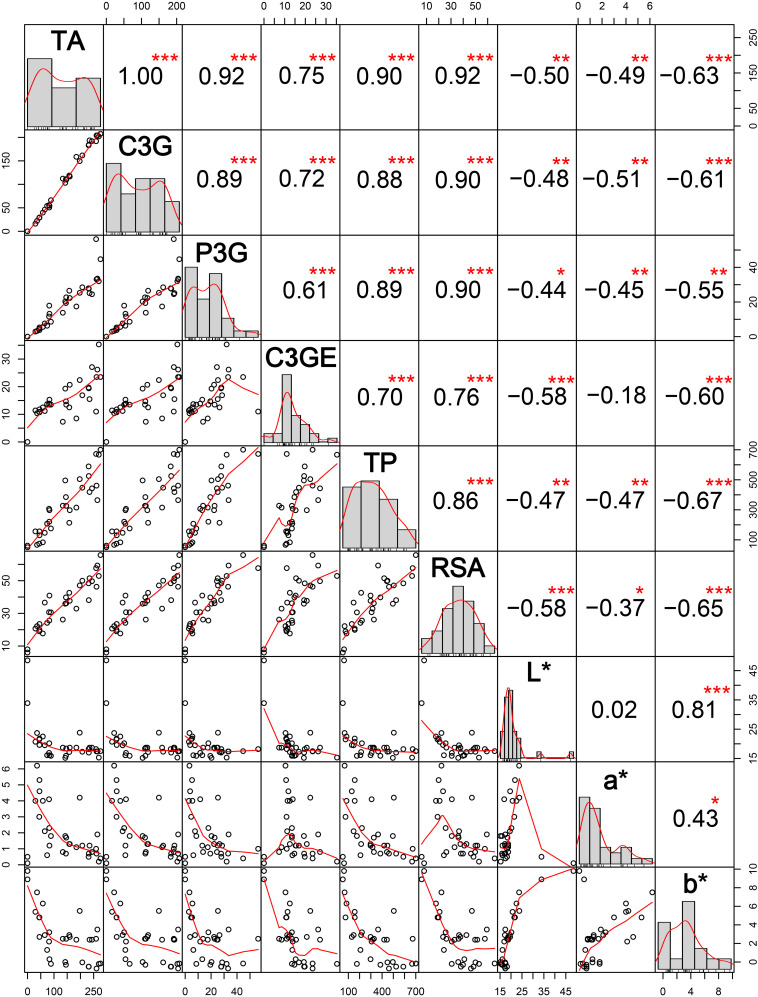
Interrelations among anthocyanin content, nutraceutical properties, and CIELAB color scales among thirty black rice genotypes from Manipur. TA, total anthocyanin content in mg 100g^– 1^ DW; C3G, cyanidin-3-o-glucoside in mg 100g^– 1^ DW; P3G, peonidin-3-o-glucoside in mg 100g^– 1^ DW; C3GE, cyanidin-3-o-glucoside equivalent in mg 100g^– 1^ DW; TP, total phenolics in mg gallic acid equivalent (GAE) 100 g^– 1^ DW; RSA, radical scavenging activity in percentage; L*, a*, and b* are CIELAB color scales. *, **, *** Significant at probability levels of 0.05, 0.01 and 0.001, respectively.

Total anthocyanin content, C3G content, P3G content, and C3GE content were significantly positively associated with total phenolics and RSA (Figure 9). Anthocyanin content was high in deep purple genotypes, mostly *Chakhao* and *Chakhao Poireiton*, as compared to dark brown or variegated purple genotypes which were pigmented on the dorsal side of the seed kernel. However, some of the deep purple genotypes (MAR 6, MAR 85) with high anthocyanin content possessed relatively lesser total phenolics as compared to lower anthocyanin content genotypes (MAR 70 and MAR 69) and *vice versa*. However, anthocyanin constituents C3G and P3G showed a positive association with RSA; some of the genotypes with equal total anthocyanin content (MAR 70, MAR 69, and MAR 36) but with a higher proportion of C3G (MAR 36) showed higher RSA (%). All the color scales (*L*^∗^, *a*^∗^, *b*^∗^) were significantly negatively associated with phytochemical parameters studied. Within color scales, *L*^∗^ depicting lightness and darkness showed variation within pigmented genotypes wherein dark purple genotypes showed values lower than 20 whereas dark/variegated brown rice possessed between 20 and 25 as compared to white rice genotypes with > 30. The *b*^∗^ value in dark purple genotypes was either negative or lower value (<3) depicting blueness (−), except *Ching Chakhao Amubi* (MAR 1 with > 3). The white and variegated brown genotypes showed a higher positive value (> 5) depicting yellowness. The value of color scale *a*^∗^ depicting redness (+) was low in white genotypes as well as deep purple genotypes and higher in dark/variegated brown genotypes. The *L*^∗^ and *b*^∗^ scales were strongly positively correlated with each other, whereas the association between *L*^∗^ and *a*^∗^ was non-significant. All the color scales were negatively associated with phytochemicals estimated.

Grouping of pigmented genotypes based on the PCA with pigmentation data identified two major PCs accounting for 83.2% of the total variation with PC1 and PC2 explaining 70 and 13% of the total variation, respectively. PC1 was majorly determined by anthocyanins (52%), phenolics (13.66%), and RSA (14.1%). PC2 was mainly influenced by color parameters *L*^∗^, *b*^∗^, and *a*^∗^, contributing 40.5, 34.2, and 9.7%, respectively. The PCA biplot clearly placed the less/non-pigmented genotypes including dark brown (1), variegated brown (4), and white (2) rice categories separately from major deep purple rice genotypes (Supplementary Figure S1).

## Discussion

Aromatic rice genotypes from north-eastern India, particularly from Manipur, are relatively lesser known cultivars because of their confined cultivation within their geographical adaptation. Recent interest on their culinary and nutraceutical properties, particularly of Manipur black rice, turned the attention of rice scientists in understanding the genetics of these genotypes. Known by their vernacular names, cultivars carrying the epithet *Chakhao*—*chak* means “rice” and *ahaoba* meaning “delicious” (Dayanidhi et al., 2017)—are a conglomeration of local landraces that share similarity in grain aroma and cooking and taste properties but genetically different. The scented glutinous *Chakhao* with black pericarp are predominantly grown in valley districts of Manipur. The black rice of Manipur has a long history of exclusive adornment of royal cuisine and highly restricted use outside the aristocracy, and their culinary properties were lesser known until recently. Recent studies on black pigmented rice in general, have established their dietary significance especially on nutraceutical properties (Goufo and Trindade, 2014; Samyor et al., 2017). Notwithstanding, the genetic identity of aromatic rices of Manipur, particularly of black rice, needs to be established to realize their potential use. Therefore, there is an immediate need to characterize their genetic diversity and to utilize them in genetic improvement, as well as to conserve them for the posterity of future generations.

Agro-morphological characterization for assessing the genetic diversity in crops draws the foundation for genetic improvement. In rice, plant height, crop duration, and grain yield are three major agronomic parameters used for determining productivity, while secondary characteristics such as panicle length, grain number, grain weight, grain color, and shape are used for classification and identification (Bhandari et al., 2017). Although limited, past research on *Chakhao* rice landraces of Manipur was carried out using fewer genotypes using fewer traits (Roy et al., 2014; Asem et al., 2015; Chanu et al., 2016). Our analyses attempt to address this lacuna, by characterizing a larger set of aromatic landraces for agro-morphological, grain cooking quality, pigmentation, and antioxidant properties as well as at the molecular level. We found no distinct association of any trait(s) with the landrace names. On the contrary, we could find that landraces sharing the same epithets differed significantly for most of the traits assessed. However, the aromatic genotypes with a deep pericarp color differed significantly for plant height, panicle length, and grain yield per plant. In general, the most widely used epithet was *Chakhao* among Manipur rice. In our study, there were 69 landraces that carried the name *Chakhao*, or its close dialectical variants such as *Chakhao* and *Chahou*. Among these, there were 12 landraces that shared the name *Chakhao Poireiton* or its close resembling name *Chakhao Poireitol*. Twenty-four genotypes, however, were only called as *Chakhao*. Further, there were ten cultivars that carried the suffix *Amubi* and seven that had *Angouba* suffixed. Prominent cultivars such as *Chakhao Poireiton* generally had strongly scented deep purple/black kernel, with tall stature, long panicles, and low grain yield. *Chakhao Angouba* cultivars were white colored with moderate scent, while *Chakhao Amubi* included all categories of pericarp color, from light brown, variegated brown, to deep purple. *Buhman* was the second prominent name among the landraces in the panel, with 16 cultivars carrying this name. While the landraces from valleys mostly carried the prefix “*Chakhao*,” the landraces from the hill districts carried the name such as “*Buhman*” (Churachandpur district), “*Maklei*” (Ukhrul district), and “*The Vumnu*” (Chandel district). Majority of the light brown pericarp color genotypes originated from hill districts. Genotypes from hill districts varied significantly from valley genotypes for their plant height and panicle length. Irrespective of the collection ecology and pericarp color, grains of the landraces were of low amylose type, with majority of them having long slender kernels. The traditional preference of the local community of Manipur is for glutinous rice with soft cooking quality. They are exclusively used in *kheer* preparation called *Chakhao Thongba*. Chanu et al. (2016) also observed low amylose in two major cultivars, namely, *Chakhao Poireiton* and *Chakhao Amubi*. They also found these two cultivars to be nutritionally rich with high dietary fiber, protein, and minerals as compared to a popular rice cultivar, Sona Mashuri, grown in southern India. Another study that evaluated 10 aromatic rice together with three non-aromatic indigenous cultivars of Manipur for grain quality revealed that most of them possessed long bold chalky grains with high ASV and low to intermediate amylose content (Thongbam et al., 2010). Our study revealed that the landraces were low yielding, exhibited high spikelet sterility, were grain shattering, and were photo-sensitive (data not presented). Since most of these landraces are conserved and grown at their respective collection sites, in order to meet the necessities of social rituals rather than for subsistence farming, there has been no serious attempt made for their systematic genetic improvement. Besides, over the decades, there has been a decline in the area of aromatic rice of Manipur with farmers opting not to grow these landraces owing to their very low yield (Borah et al., 2018). If this trend continues, it might lead to genetic erosion of these valuable indigenous landraces. Alternatively, because of the benefits such as nutraceutical properties, pigmentation, glutinous endosperm, and aroma, pigmented rice is gaining popularity among the consumers across the world. Additionally, we found that some of the landraces also possessed desirable traits such as early maturity and relatively higher grain yield, which would provide ample scope for crop improvement. For systematic improvement of Manipur aromatic rice, for both yield and quality, it is desirable to generate a profile of desirable traits within this gene pool, such as kernel pigmentation and antioxidant properties as well as undesirable traits such as photosensitivity, seed shattering, low spikelet fertility, and poor yield.

Analysis of genome-wide variations using molecular markers is one of the means to delineate the evolutionary relationship between genotypes that are believed to share a common ancestry. SSRs are highly useful in assessing diversity among closely related rice cultivars (Singh et al., 2013; Yadav et al., 2013) as they provide better resolvability, are multi-allelic, provide genome-wide coverage, are highly reproducible, are easy to score, and are cost-effective (Akagi et al., 1997; Singh and Singh, 2015). There are several studies that used SSR markers for finding the genetic structure of the rice germplasm, as one of the most widely used molecular markers for genetic diversity studies (Nachimuthu et al., 2015; Singh et al., 2016; Aljumaili et al., 2018; Islam et al., 2018a, b; Pathaichindachote et al., 2019; Suvi et al., 2019; Verma et al., 2019). Further, SSR markers are also proven to be efficient in delineating major genetic groups of rice, namely, *indica*, temperate *japonica*, tropical *japonica*, *aus*, and aromatic (Roy et al., 2015, 2016; Wang et al., 2014). In the present study, the panel of 48 SSR markers used could establish the diversity pattern within the Manipur aromatic rice germplasm. Although the gene diversity (0.443) as well as the PIC value (0.394) was lower than those observed from previous studies (Das et al., 2013), this could be attributed to the narrow ecological range from which the lines were sourced, as well as to a greater number of genotypes tested in this study. Notwithstanding, a significant level of genetic variation observed within the lines could be due to their long history of evolution in specific ecologies. An attempt was made in the present study to classify the aromatic rice landraces into rice ecotypes *indica*, *tropical* and *temperate japonica*, and *aus* and their admixtures based on molecular data. The germplasm contained three subpopulations with the checks distributed in the first two subpopulations. As expected, *indica* genotypes (POP2) dominated *japonica* types (POP1) by number and possessed more admixtures. The hilly accessions were more of *japonica* type (POP1) and had very less admixtures. This indicated that even as there was population pressure coming from the majority of *indica* subtypes, the *japonica* types retained their genetic identity among the Manipur aromatic germplasm. Earlier, Roy et al. (2016) reported the predominance of *japonica* and their admixtures among the hill rice of northeast India. POP2 included genotypes such as MAR16, MAR51, MAR54, MAR57, MAR58, MAR68, and MAR98, which possessed most of the rare and unique alleles from both hill and valley regions. Pigmented rice formed a separate large subpopulation (POP3) among the genotypes tested. The origin of black rice of Manipur could be related to introduction of *japonica* rice from China, beginning from second century BC (Tensuba, 1993; Singh and Baghel, 2003; Lalit, 2007). Prominent among the members of POP3 were *Chakhao* landraces that included both pigmented and unpigmented types. Our study also revealed that the pigmented rice showed significant variation in the content of phytochemicals such as anthocyanin and phenolics, implicating that the *Chakhao* landraces had undergone isolated conservation in the local farm holds. As the RSA was found highly correlated with color parameters, these lines also possessed high antioxidant properties in the rice grain.

Earlier, genome analysis of 21 black rice landraces demonstrated that the origin of black rice gene occurred within tropical *japonica*, which later migrated to *indica* and then to temperate *japonica* (Oikawa et al., 2015). Signature of cross subspecific migration of black rice trait could also be seen in the present study, wherein one landrace *Maklei* (dark brown) from the hill district of Ukhrul was grouped along with *japonica* type, whereas the remaining black rice landraces were grouped within the *indica* subgroup cluster. However, this observation needs to be further confirmed by taking more reference entries of respective subgroups and evaluating the diversity with high-density genome-wide markers. Further, reinforcing the theory of cross subspecific migration, based on population structure, we could identify a subpopulation (POP3) with predominance in genotypes with deep purple pericarp as well as for hill and valley adapted genotypes. However, Roy et al. (2014) reported six subgroups such as *Chakhao Poireiton*, *Chakhao Amubi*, *Chakhao, Maklei*, *Buhman*, and *Chakhao Angouba* in 37 *Chakhao* landraces based on 47 random SSR markers, which could be due to a limited number of genotypes used in the study.

Rice pigmentation is attributed to the accumulation of anthocyanins in the pericarp of the grains. Anthocyanins are subgroups of flavonoids which are water-soluble pigments imparting different shades of red, blue, purple, to plant parts. They belong to a class of secondary metabolites of the polyphenol group. In the present study, C3G and P3G were found to be the predominant anthocyanin compounds, while the unidentified fraction was quantified as C3GE compounds. The identification was based on the mass spectral values depicted as m/z ratio. The aglycones, cyanidin and peonidin, have a m/z value of 287.05 and 301.07, respectively (Abdel-Aal et al., 2006; Kim et al., 2008; Lee, 2010), which, on addition of the sugar moiety attachment of glucose (m/z: 162.00), makes the total m/z to 449.1 and 463.1 corresponding to C3G and P3G. In our spectrum, corresponding signals were identified as the prominent peaks confirming the presence of C3G and P3G. The presence of both fractionated and unfractionated residues, served as a reconfirmation of the result. Identification of similar compounds was earlier reported from the black rice cultivars, *Heugjinjubyeo* (Lee, 2010) and *Kilimheugmi* (Ryu et al., 1998) from Korea and black rices of China (Sompong et al., 2011). Hou et al. (2013) identified the constituents of the C3GE fraction from the *japonica* black rice variety, Longjing No. 1 of China, as cyanidin 3,5-diglucoside and cyanidin-3-rutinoside. Among the Japanese black rice, Chen et al. (2012) identified four different anthocyanins such as C3G, P3G, malvidin, and petunidin-3-O-glucoside (Pt-3G). Asem et al. (2015) reported delphinidin 3-galactoside, delphinidin 3-arabinoside, cyanidin 3-galactoside, and C3G from *Chakhao Poireiton*. Further, they had also identified the first three of these compounds in *Chakhao Amubi*. However, the identification of these compounds in their study was based on the comparison of retention time (RT) reported in earlier publications, which is very subjective in nature leading to a possibility of errors due to changes in the solvent system and the instrumentation conditions of the assessment.

The total anthocyanins among the rice landraces in the present study varied widely, imparting different color shades to the kernels. It is interesting to note that genotypes sharing the same epithet(s) as a part of their names like *Chakhao Poireiton*, *Chakhao Amubi*, and *Ching Chakhao* but which were collected from different localities within Manipur significantly varied for total anthocyanin content and its constituents. There was no significant difference in patterns between genotypes from hill districts and valley districts. Although anthocyanin content is reported to be influenced by the environment (Somsana et al., 2013), in the present study, the genotypes were grown uniformly in the experimental plots at a valley region (Imphal), and hence, the variation among the different lines could be purely genetical. Further, pigmentation had no influence on the genotype adaptation to different ecologies. However, any influence of pericarp development, seed coat thickness, grain shape, and weight on the anthocyanin accumulation and color intensity among the pigmented rice of Manipur needs further detailed investigations. Shen et al. (2009) have reported that flavonoid and phenolic contents were positively related to grain shape and negatively related to grain weight. Likewise, we could also observe that the intensity of the pigmentation depended on the proportion and content of pigment compounds, starting from light color to dark. Deeply pigmented rice kernels rather appear black and are popularly traded as black or purple rice. The nutraceutical properties of the pigmented rice and their potential health benefits have been established using cell lines, animal models, and human clinical trials (Pojer et al., 2013; Das et al., 2014; Samyor et al., 2017; Khoo et al., 2017; Seechamnanturakit et al., 2018; Thanuja and Parimalavalli, 2018; Callcott et al., 2019; Limtrakul et al., 2019).

Traditionally, purple or black rice is grown and consumed in many Asian countries like China, Thailand, Sri Lanka, Republic of Korea, Vietnam, Indonesia, India, Philippines, and Japan. In India, popular black rice varieties include *Chakhao* rice from Manipur and *Kalabhat* from West Bengal. The anthocyanin content of black rice from Korea was as high as 493 mg.100g^–1^, having a large fraction of C3G varying between 80 and 95.3% (Ryu et al., 1998), while Lee (2010) reported C3G content ranging from 52.1 ± 6.3 to 1601.0 ± 8.5 μg.g^–1^ and P3G content from 0.0 to 82.6 ± 1.2 μg.g^–1^. Anthocyanins being the constituent of polyphenols in plants, the total phenolic content show a significant correlation with anthocyanin content, particularly in pigmented rice (Dai and Mumper, 2010; Deng et al., 2013).

The radical scavenging property of antioxidant compounds provides immense health benefits by scavenging reactive oxygen species (ROS) and other harmful free radicals when oxidative stress occurs (Dröge, 2002). When cellular machinery fails to contain the stress, antioxidants such as anthocyanins can prevent damage either by delaying the oxidative process or by scavenging the excess free radicals. However, antioxidant property differs in different anthocyanins depending upon their molecular structure. The activity increases with the increased number of free hydroxyl group around the pyrone ring (Miguel, 2011). Among the most common anthocyanins, cyanidin is the most active against superoxide after delphinidin. In the present study, C3G was found to be the predominant anthocyanin pigment in *Chakhao* landraces, which could play an important role in scavenging the superoxides. The RSA of the pigmented rice was significantly higher than that of unpigmented genotypes, ranging from more than two to eight times. Chanu et al. (2016) reported an RSA of 72.5% in *Chakhao Poireiton* and 59.0% in *Chakhao Amubi*. Although RSA identified in this study was slightly less than these values, we could observe a maximum activity of 65.7% in our panel. However, the presence of a large variation in free RSA *vis-à-vis* total anthocyanin observed in different deep purple genotypes with similar epithets necessitates defining the baseline values for grain quality and nutraceutical standards aiding improvement and promotion of this speciality rice.

## Conclusion

In the present study, we elucidated a large variation for the agro-morphological, grain quality, pigmentation, and phytochemical characteristics in the hitherto uncharacterized aromatic rice landraces of Manipur. Although several of them shared common epithets, we found that they are significantly diverse and shared distinct subpopulation memberships based on genetic data. Despite the commonness in names, the average gene diversity of the landraces was 0.443, implying their genetic uniqueness. This necessitates efforts for conservation, documentation, and utilization of this diversity for further improvement. The striking feature of Manipur aromatic rice is the variation for pericarp pigmentation ranging from white to dark purple/black color. Several of the pigmented genotypes are low in amylose with soft cooking quality and high anthocyanin content. Two growing niches in the region *viz*., valley and hill, also show a distinct pattern of genotype characteristics. A detailed investigation with high-throughput markers such as single nucleotide polymorphisms (SNPs) providing high-density genome-wide coverage can help in assessing the genetic relatedness of these genotypes with *indica* and *japonica* subtypes, as well as for mining novel allelic variants that may be present in this gene pool. There is also a need to assess grain quality through metabolic profiling, quantification of aroma, and micronutrient content, which can lay a strong foundation for *in situ* conservation and improvement of these unique landraces from Manipur.

## Data Availability Statement

All datasets presented in this study are included in the article/Supplementary Material.

## Author Contributions

AS and SG conceptualized the idea and supervised the experiments. SB, SG, and NS: carried molecular work. SB, HB, and SS carried and monitored quality, phytochemical estimations. RE, KV, and SB carried out the statistical analysis. SB, IS, NP, PB, and MN carried and monitored field experiments. SB, KV, and SG prepared manuscript. All authors read and approved the final manuscript.

## Conflict of Interest

The authors declare that the research was conducted in the absence of any commercial or financial relationships that could be construed as a potential conflict of interest.

## Footnotes

1 http://ipindiaservices.gov.in/GirPublic/Application/Details/602
