# Supplementary material for: Genetic Architecture and Anthocyanin Profiling of Aromatic Rice From Manipur Reveals Divergence of Chakhao Landraces
Source: Front Genet. 2020 Oct 15;11:570731. doi: 10.3389/fgene.2020.570731 (PMC7593561; doi:10.3389/fgene.2020.570731)
Supplement: Supplementary file 1 [file Data_Sheet_1.pdf]

frontiers in Genetics

Supplementary information

Genetic Architecture and Anthocyanin Profiling of Aromatic Rice from Manipur Reveals Divergence of *Chakhao* Landraces

S. Bhuvaneswari[1,2], S. Gopala Krishnan[1], Haritha Bollinedi[1], Supradip Saha[3], Ranjith Kumar Ellur[1], K. K. Vinod[1], I. Meghachandra Singh[3], Narendra Prakash[3], Prolay Kumar Bhowmick[1], M. Nagarajan[4], Nagendra Kumar Singh[5] and Ashok Kumar Singh[1,*]

[1] Division of Genetics, ICAR-Indian Agricultural Research Institute, New Delhi, India, [2] ICAR-Regional Centre for North-Eastern Hill Region, Manipur Centre, Imphal, India, [3] Division of Agricultural Chemicals, ICAR-Indian Agricultural Research Institute, New Delhi, India, [4] Rice Breeding and Genetics Research Centre, ICAR-Indian Agricultural Research Institute, Aduthurai, India, [5] ICAR-National Institute of Plant Biotechnology, New Delhi, India

**Corresponding author: aks_gene@yahoo.com*

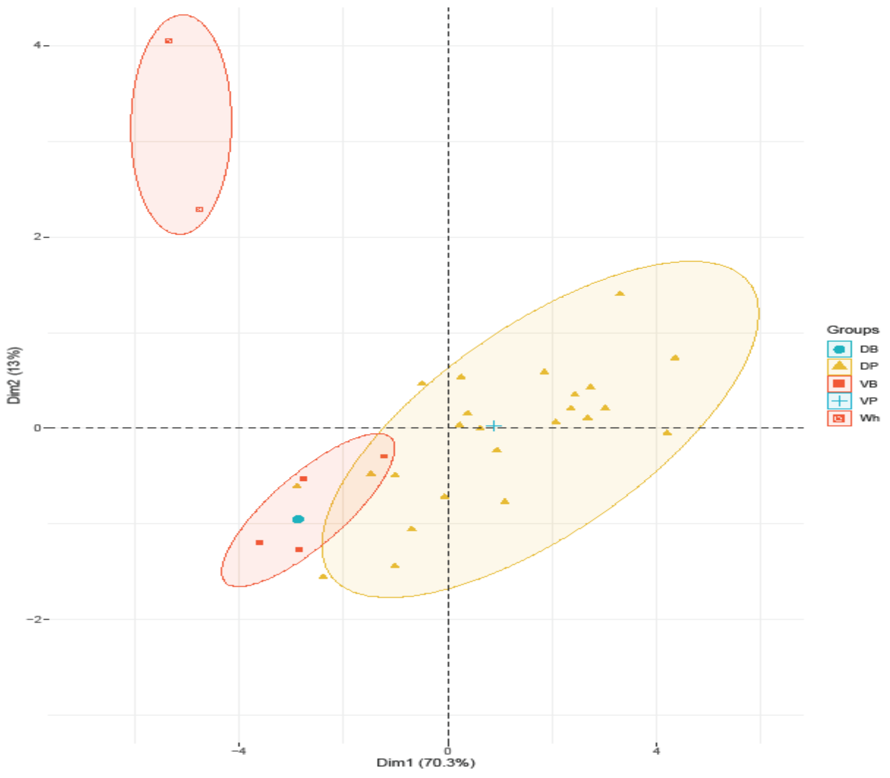

Supplementary Figure S1. Principal Component Analysis biplot of *Chakhao* rice genotypes from Manipur.

Bhuvaneswari S, Gopala Krishnan S, Bollinedi H, Saha S, Ellur RK, Vinod KK, Singh IM, Prakash N, Bhowmick PK, Nagarajan M, Singh NK and Singh AK (2020) Genetic Architecture and Anthocyanin Profiling of Aromatic Rice From Manipur Reveals Divergence of Chakhao Landraces. Front. Genet. 11:570731.doi: 10.3389/fgene.2020.570731

Supplementary Table S1. The aromatic rice germplasm from Manipur used in the present study

Code	Accession No.	Local Name	District	Ecosystem	Pericarp colour	Aroma
MAR1	IC0306333	*Ching Chakhao Amubi*	Imphal east	Valley	Dark purple	SS
MAR2	IC0461320	*Chakhao*	NA	NA	White	MS
MAR3	IC0461323	*Chakhao Angenba*	NA	NA	White	MS
MAR4	IC0461895	*Chakhao Poireitol*	NA	NA	White	MS
MAR5	IC0461922	*Chakhao Poiretol*	NA	NA	White	MS
MAR6	IC0464571	*Chakhao Poireitol*	Thoubal	Valley	Dark purple	MS
MAR7	IC0464574	*Chakhao Poireitol*	Thoubal	Valley	Dark purple	SS
MAR8	IC0464578	*Chakhao*	Thoubal	Valley	White	MS
MAR9	IC0464580	*Chakhao*	Manipur Central	Valley	White	NS
MAR10	IC0464583	*Chakhao*	Manipur Central	Valley	Dark purple	SS
MAR11	IC0464585	*Chakhao*	Manipur Central	Valley	White	NS
MAR12	IC0464590	*Chakhao*	Thoubal	Valley	Light brown	SS
MAR15	IC0465318	*Chakhao*	Imphal west	Valley	Dark purple	SS
MAR16	IC0465365	*Chakhao Arongbi*	Thoubal	Valley	White	MS
MAR17	IC0465376	*Chakhao*	Manipur Central	Valley	Dark brown	MS
MAR18	IC0467090	*Kabo Chakhao*	Imphal East	Valley	Light brown	MS
MAR19	IC0467095	*Chakhao Angouba*	Imphal East	Valley	White	NS
MAR21	IC0467110	*Kabo Chakhao*	Manipur Central	Valley	White	MS
MAR22	IC0467147	*Chakhao Sempak*	Imphal East	Valley	Light brown	MS
MAR24	IC0467155	*Chakhao*	Manipur Central	Valley	Light brown	MS
MAR25	IC0467157	*Chakhao*	Manipur Central	Valley	Light brown	MS
MAR26	IC0467169	*Chakhao Poireitol*	Thoubal	Valley	White	MS
MAR27	IC0467170	*Chakhao*	Manipur Central	Valley	Light brown	MS
MAR28	IC0467175	*Chakhao*	Thoubal	Valley	White	SS
MAR30	IC0596559	*Chakhao Poireiton*	Imphal west	Valley	Dark purple	MS
MAR31	IC0596560	*Chakhao Angouba*	Imphal west	Valley	White	NS
MAR32	IC0596564	*Chakhao*	Senapati	Valley	White	NS
MAR33	IC0596565	*Chakhao Poireiton*	Thoubal	Valley	Dark purple	MS
MAR34	IC0596566	*Chakhao Amubi*	Thoubal	Valley	Dark purple	NS
MAR35	IC0596567	*Chakhao Poireiton*	Thoubal	Valley	Dark purple	MS
MAR36	IC0596568	*Chakhao Pungdol Angouba*	Thoubal	Valley	Dark purple	MS
MAR37	IC0596569	*Chakhao Poireiton*	Thoubal	Valley	White	SS
MAR38	IC0596570	*Chakhao Amubi*	Thoubal	Valley	White	NS
MAR39	IC0596571	*Chakhao Angouba*	Thoubal	Valley	White	NS
MAR40	IC0596573	*Chakhao Amubi*	Chandel	Hill	Variegated brown	SS
MAR41	IC0596574	*Chakhao Angouba*	Chandel	Hill	White	MS
MAR42	IC0596585	*Chakhao Phou*	Tamenglong	Hill	Light brown	MS
MAR43	IC0596586	*Chakhao Phou*	Tamenglong	Hill	White	-
MAR44	IC0596587	*Chakhao Poireiton*	Bishnupur	Valley	Dark purple	MS
MAR45	IC0596588	*Chakhao*	Bishnupur	Valley	Dark purple	NS
MAR46	IC0596589	*Chakhao Amubi*	Bishnupur	Valley	Variegated brown	MS
MAR47	IC0596590	*Chakhao Poireiton*	Bishnupur	Valley	White	NS
MAR48	IC0350432	*Buhman*	Churachandpur	Hill	White	MS
MAR49	IC0350433	*Buhman - A*	Churachandpur	Hill	Light brown	MS
MAR50	IC0350434	*Buhman Kungtum*	Churachandpur	Hill	Light brown	MS
MAR51	IC0350436	*Buhman Te*	Churachandpur	Hill	Light brown	NS
MAR52	IC0350437	*Buhman Tum*	Churachandpur	Hill	Light brown	MS
MAR53	IC0350588	*Sang Buhman*	Churachandpur	Hill	Light brown	MS
MAR54	IC0591271	*Buhman*	Churchandpur	Hill	Light brown	MS
MAR55	IC0591272	*Sang Buhman*	Churchandpur	Hill	White	NS
MAR56	IC0596576	*Buhman (Ch. Angouba)*	Churachandpur	Hill	White	NS
MAR57	IC0596577	*Buhman*	Churachandpur	Hill	Light brown	MS
MAR58	IC0596578	*Buhman*	Churachandpur	Hill	Light brown	MS
MAR59	IC0596579	*Buhman*	Churachandpur	Hill	White	MS
MAR60	IC0596580	*Buhman*	Churachandpur	Hill	Light brown	MS
MAR61	IC0596581	*Buhman/ Idaw*	Churachandpur	Hill	Light brown	MS

Code	Accession No.	Local Name	District	Ecosystem	Pericarp colour	Aroma
MAR62	IC0596582	*Buhman*	Churachandpur	Hill	White	NS
MAR63	IC0596591	*Maklei*	Ukhrul	Hill	Variegated brown	MS
MAR64	IC0596592	*Maklei*	Ukhrul	Hill	Dark purple	MS
MAR65	IC0596593	*Maklei*	Ukhrul	Hill	Light brown	MS
MAR66	IC0596594	*Maklei*	Ukhrul	Hill	Light brown	MS
MAR67	IC0596583	*Napnang Hangmei*	Tamenglong	Hill	White	MS
MAR68	IC0596584	*Napnang Hangmei*	Tamenglong	Hill	Light brown	SS
MAR69	SB/PB 1	*Chakhao Amubi*	Imphal East	Valley	Dark purple	MS
MAR70	SB/SKS 1	*Chakhao Poireiton*	Imphal East	Valley	Dark purple	SS
MAR75	SB/LM 1	*The Vumnu*	Chandel	Hill	Dark purple	SS
MAR76	SB/LM 2	*The Vumnu*	Chandel	Hill	Dark purple	MS
MAR77	SB/LM 3	*Ethe Buw*	Chandel	Hill	Variegated brown	MS
MAR78	SB/SS 1	Black rice	Imphal east	Valley	Variegated Purple	MS
MAR79	SB/IMS 1	*Buhman*	Churachandpur	Hill	Dark purple	MS
MAR80	SB/IMS 2	*Chakhao Kumbi*	NA	Valley	Light brown	MS
MAR81	SB/IMS 3	*Chakhao Poireiton*	NA	Valley	White	SS
MAR82	SB/IMS 4	*Chakhao Amubi*	NA	Valley	Dark purple	SS
MAR83	SB/PB 2	*Langphou Chakhao*	NA	Valley	Dark purple	MS
MAR84	SB/IMS 5	*Chakhao*	Tamenglong	Hill	Light brown	MS
MAR85	SB/RKY 1	*Chakhao*	Ukhrul	Hill	Dark purple	SS
MAR86	SB/CT 1	*Ching Chakhao*	Imphal east	Valley	Dark purple	SS
MAR87	IC0352915	*Chakhao Poireiton*	Thoubal	Valley	Variegated brown	MS
MAR88	IC0467085	*Chakhao*	Imphal East	Valley	White	MS
MAR89	IC0467146	*Chahou Poireiton*	Thoubal	Valley	White	MS
MAR91	IC0521521	*Chakhao*	Imphal	Valley	Dark purple	SS
MAR93	IC0467102	*Kabo Chakhao*	Manipur Central	Valley	White	MS
MAR94	IC0350440	*Chakhao Amubi*	NA	Valley	White	MS
MAR97	IC0352872	*Chakhao Amuba*	Imphal East	Valley	Dark purple	SS
MAR98	IC0352914	*Chakhao Angangbi*	Thoubal	Valley	White	MS
MAR99	IC0352917	*Chakhao Huikap*	Thoubal	Valley	Dark Purple	SS
MAR101	IC0591277	*Chakhao*	East Imphal	Valley	Dark Purple	MS
MAR102	IC0591278	*Chakhao*	East Imphal	Valley	White	SS
MAR103	IC0306379	*Chakhao Amubi*	Bishnupur	Valley	Variegated Purple	SS
MAR105	IC0306382	*Chakhao Poireiton*	Bishnupur	Valley	White	SS
MAR106	IC0350441	*Chahao Angouba*	NA	Valley	Light brown	MS
MAR108	IC0297667	*Chakhao*	Imphal East	Valley	Light Brown	MS
MAR109	IC0306383	*Chakhao Angouba*	Bishnupur	Valley	White	MS

SS, strongly scented; MS, mild scented; NS, unscented; NA, data not available

Supplementary Table S2. Principal component analysis of agronomic and grain quality data a showing most significantly contributing traits (data shows first ten principal components)

Statistics	PC1	PC2	PC3	PC4	PC5	PC6	PC7	PC8	PC9	PC10
Standard deviation	2.31	1.58	1.32	1.12	1.07	0.99	0.94	0.80	0.73	0.68
Proportion of Variance	0.31	0.15	0.10	0.07	0.07	0.06	0.05	0.04	0.03	0.03
Cumulative Proportion	**0.31**	**0.46**	**0.56**	**0.64**	**0.70**	0.76	0.81	0.85	0.88	0.91
Eigenvalues	**5.32**	**2.50**	**1.73**	**1.25**	**1.14**	0.98	0.88	0.64	0.53	0.46
Ligule colour (LG)	**-0.41**	0.04	0.05	-0.07	-0.08	0.08	-0.14	0.05	-0.09	0.01
Auricle colour (AU)	**-0.39**	0.07	0.11	-0.04	-0.08	0.01	-0.06	-0.07	0.11	0.07
Collar colour (CO)	**-0.39**	0.04	0.05	-0.08	-0.03	0.00	-0.12	-0.17	0.10	-0.03
Basal leaf colour (BL)	**-0.33**	0.07	0.06	-0.12	-0.13	-0.03	0.09	-0.08	0.71	-0.07
Grain colour (LP)*	**-0.38**	0.08	0.03	-0.02	0.07	0.05	0.06	0.06	-0.31	-0.09
Pericarp colour (PC)	**-0.38**	0.04	0.09	-0.07	-0.03	0.11	-0.08	0.12	-0.36	-0.17
Flowering days (DF)	-0.06	-0.29	0.11	-0.27	-0.42	-0.47	0.02	0.48	-0.07	0.41
Plant height (PH)	-0.19	**-0.41**	0.18	0.11	0.27	0.05	0.21	-0.01	-0.25	-0.08
Panicle number (PN)	-0.07	-0.31	-0.12	-0.24	0.50	-0.29	-0.31	-0.33	0.09	0.32
Panicle length (PL)	-0.10	-0.22	0.32	0.39	0.40	0.20	0.20	0.33	0.25	0.29
Grain weight (GW)	0.09	-0.17	0.52	0.05	-0.25	-0.08	-0.01	-0.63	-0.17	0.09
Grain yield (GY)	0.15	-0.26	0.22	-0.48	0.24	-0.03	-0.21	0.21	0.12	-0.59
Kernel length (KL)	0.08	-0.21	0.12	0.32	-0.24	0.31	-0.77	0.14	0.09	0.02
Kernel width (KW)	0.17	0.06	0.57	-0.11	-0.11	0.05	0.23	0.05	0.09	-0.11
Alkali spreading (AS)	-0.10	-0.07	-0.03	0.57	-0.03	-0.66	-0.04	0.01	0.06	-0.44
Gel consistency (GC)	0.07	**0.44**	0.27	-0.04	0.28	-0.25	-0.24	0.15	-0.18	0.14
Amylose content (AC)	-0.01	**-0.49**	-0.27	-0.03	-0.19	0.15	0.17	-0.04	-0.07	-0.08

*Pigmentation of lemma and palea; The most contributing traits are indicated in red bold

Bhuvaneswari S, Gopala Krishnan S, Bollinedi H, Saha S, Ellur RK, Vinod KK, Singh IM, Prakash N, Bhowmick PK, Nagarajan M, Singh NK and Singh AK (2020) Genetic Architecture and Anthocyanin Profiling of Aromatic Rice From Manipur Reveals Divergence of Chakhao Landraces. Front. Genet. 11:570731.doi: 10.3389/fgene.2020.570731

Supplementary Table S3. Major allele frequency, allele number, gene diversity and polymorphic information content (PIC) of SSR markers used in the study

SSR marker	Chr	Major allele frequency	Number of alleles	Gene diversity	PIC
RM495	1	0.557	2	0.494	0.372
RM1	1	0.835	5	0.293	0.280
RM283	1	0.602	3	0.502	0.403
RM259	1	0.660	4	0.500	0.442
RM312	1	0.888	2	0.199	0.179
RM5	1	0.567	4	0.538	0.447
RM237	1	0.786	3	0.360	0.331
RM431	1	0.633	4	0.492	0.406
RM154	2	0.367	4	0.671	0.600
RM452	2	0.526	3	0.517	0.403
RM489	3	0.856	3	0.250	0.223
OSR13	3	0.704	3	0.433	0.362
RM338	3	0.867	2	0.230	0.204
RM55	3	0.786	3	0.355	0.322
RM514	3	0.713	3	0.438	0.383
RM307	4	0.408	6	0.733	0.694
RM124	4	0.898	2	0.183	0.166
RM507	5	0.831	3	0.284	0.249
RM413	5	0.316	7	0.774	0.741
RM161	5	0.889	2	0.198	0.178
RM178	5	0.908	3	0.168	0.157
RM334	5	0.446	6	0.646	0.577
RM510	6	0.735	3	0.425	0.386
RM162	6	0.786	3	0.341	0.289
RM454	6	0.959	2	0.078	0.075
RM125	7	0.939	2	0.115	0.108
RM11	7	0.704	5	0.462	0.419
RM455	7	0.867	2	0.230	0.204
RM118	7	0.755	2	0.370	0.301
RM408	8	0.418	4	0.661	0.591
RM152	8	0.418	4	0.654	0.587
RM25	8	0.629	5	0.558	0.520
RM44	8	0.800	3	0.340	0.313
RM284	8	0.885	2	0.203	0.182
RM433	8	0.515	2	0.500	0.375
RM447	8	0.755	4	0.400	0.365
RM316	9	0.737	4	0.425	0.389
RM105	9	0.469	3	0.587	0.498
RM215	9	0.753	3	0.401	0.364
RM474	10	0.560	6	0.624	0.583
RM271	10	0.531	4	0.644	0.599
RM171	10	0.804	3	0.322	0.282
RM552	11	0.206	7	0.826	0.802
RM536	11	0.857	3	0.252	0.233
RM287	11	0.427	4	0.632	0.560
RM144	11	0.306	7	0.785	0.753
RM19	12	0.541	4	0.614	0.556
RM277	12	0.495	3	0.555	0.455
**Mean**		**0.664**	**3.563**	**0.443**	**0.394**

Bhuvaneswari S, Gopala Krishnan S, Bollinedi H, Saha S, Ellur RK, Vinod KK, Singh IM, Prakash N, Bhowmick PK, Nagarajan M, Singh NK and Singh AK (2020) Genetic Architecture and Anthocyanin Profiling of Aromatic Rice From Manipur Reveals Divergence of Chakhao Landraces. Front. Genet. 11:570731.doi: 10.3389/fgene.2020.570731

Chr, chromosome; PIC, polymorphism information content

Supplementary Table S4. Retention time of different peaks found in the anthocyanin rich black rice extract (ABRE) of samples and known standards

	**Peak 1**	**Peak 2**	**Peak 3**	**Peak 4**
Retention time (min)	10.97	11.56	11.95	12.9
Compounds	Unknown	Unknown	Cyanidin-3-O-glucoside	Peonidin-3-O-glucoside

Bhuvaneswari S, Gopala Krishnan S, Bollinedi H, Saha S, Ellur RK, Vinod KK, Singh IM, Prakash N, Bhowmick PK, Nagarajan M, Singh NK and Singh AK (2020) Genetic Architecture and Anthocyanin Profiling of Aromatic Rice From Manipur Reveals Divergence of Chakhao Landraces. Front. Genet. 11:570731.doi: 10.3389/fgene.2020.570731
